# An Integrative Analysis of DNA Methylation Pattern in Myotonic Dystrophy Type 1 Samples Reveals a Distinct DNA Methylation Profile between Tissues and a Novel Muscle-Associated Epigenetic Dysregulation

**DOI:** 10.3390/biomedicines10061372

**Published:** 2022-06-10

**Authors:** Emma Koehorst, Renato Odria, Júlia Capó, Judit Núñez-Manchón, Andrea Arbex, Miriam Almendrote, Ian Linares-Pardo, Daniel Natera-de Benito, Verónica Saez, Andrés Nascimento, Carlos Ortez, Miguel Ángel Rubio, Jordi Díaz-Manera, Jorge Alonso-Pérez, Giuseppe Lucente, Agustín Rodriguez-Palmero, Alba Ramos-Fransi, Alicia Martínez-Piñeiro, Gisela Nogales-Gadea, Mònica Suelves

**Affiliations:** 1 Neuromuscular and Neuropediatric Research Group, Institut d’Investigació en Ciències de la Salut Germans Trias i Pujol (IGTP), Campus Can Ruti, Universitat Autònoma de Barcelona, 08916 Badalona, Spain; ekoehorst@igtp.cat (E.K.); rrodria@igtp.cat (R.O.); jcapo@igtp.cat (J.C.); jnunez@igtp.cat (J.N.-M.); aarbexb.germanstrias@gencat.cat (A.A.); malmendrote.germanstrias@gencat.cat (M.A.); ilinares@igtp.cat (I.L.-P.); glucente@igtp.cat (G.L.); arodriguezpalmero.germanstrias@gencat.cat (A.R.-P.); aramosf@igtp.cat (A.R.-F.); amartinezp.germanstrias@gencat.cat (A.M.-P.); gnogales@igtp.cat (G.N.-G.); 2 Neuromuscular Pathology Unit, Neurology Service, Neuroscience Department, Hospital Universitari Germans Trias i Pujol, 08916 Badalona, Spain; 3 Neuromuscular Unit, Neuropediatric Department, Institut de Recerca Pediàtrica Hospital Sant Joan de Déu, L’Hospitalet de Llobregat, 08950 Barcelona, Spain; daniel.natera@sjd.es (D.N.-d.B.); veroisabelsaez@gmail.com (V.S.); anascimento@sjdhospitalbarcelona.org (A.N.); ciortez@sjdhospitalbarcelona.org (C.O.); 4 Neuromuscular Unit, Department of Neurology, Hospital del Mar, 08003 Barcelona, Spain; marubio@psmar.cat; 5 Neuromuscular Diseases Unit, Department of Neurology, Hospital de la Santa Creu i Sant Pau, 08025 Barcelona, Spain; jordi.diaz-manera@newcastle.ac.uk (J.D.-M.); jalonsop@santpau.cat (J.A.-P.); 6 John Walton Muscular Dystrophy Research Centre, Newcastle University and Newcastle Hospitals NHS Foundation Trust, Newcastle upon Tyne NE1 3BZ, UK; 7 Pediatric Neurology Unit, Department of Pediatrics, Hospital Universitari Germans Trias i Pujol, Universitat Autònoma de Barcelona, 08916 Badalona, Spain

**Keywords:** myotonic dystrophy, CpG islands, DNA methylation, epigenetics, phenotype severity, DM1 biopsies, cellular models

## Abstract

Myotonic dystrophy type 1 (DM1) is a progressive, non-treatable, multi-systemic disorder. To investigate the contribution of epigenetics to the complexity of DM1, we compared DNA methylation profiles of four annotated CpG islands (CpGis) in the *DMPK* locus and neighbouring genes, in distinct DM1 tissues and derived cells, representing six DM1 subtypes, by bisulphite sequencing. In blood, we found no differences in CpGi 74, 43 and 36 in DNA methylation profile. In contrast, a CTCF1 DNA methylation gradient was found with 100% methylation in congenital cases, 50% in childhood cases and 13% in juvenile cases. CTCF1 methylation correlated to disease severity and CTG expansion size. Notably, 50% of CTCF1 methylated cases showed methylation in the CTCF2 regions. Additionally, methylation was associated with maternal transmission. Interestingly, the evaluation of seven families showed that unmethylated mothers passed on an expansion of the CTG repeat, whereas the methylated mothers transmitted a contraction. The analysis of patient-derived cells showed that DNA methylation profiles were highly preserved, validating their use as faithful DM1 cellular models. Importantly, the comparison of DNA methylation levels of distinct DM1 tissues revealed a novel muscle-specific epigenetic signature with methylation of the CTCF1 region accompanied by demethylation of CpGi 43, a region containing an alternative *DMPK* promoter, which may decrease the canonical promoter activity. Altogether, our results showed a distinct DNA methylation profile across DM1 tissues and uncovered a novel and dual epigenetic signature in DM1 muscle samples, providing novel insights into the epigenetic changes associated with DM1.

## 1. Introduction

Myotonic dystrophy type 1 (DM1) is an autosomal dominant inherited, multi-systemic disorder, with predominant muscle involvement and an estimated incidence of 1:8000 [1]. DM1 has been recognized as one of the muscle dystrophies with the more variable phenotype, as it affects several tissues and systems, and because it has varied manifestations. It can be classified into five different clinical subtypes, which are based on the age of onset, ranging from foetal to late-adult onset [2]. These clinical subtypes show an increasing disease severity with decreasing age of onset, and although they share a core set of symptoms, each subtype has unique additional features (Table 1) [2,3,4,5,6,7,8,9,10,11,12]. In addition to the five clinical categories, there is another special set of ‘DM1’ patients, the asymptomatic or paucisymptomatic DM1 category, characterized by the absence or just minor symptoms across an individual’s life span. The causes of the clinical variability observed in DM1 are poorly understood.

Epigenetics is defined as heritable changes that do not affect the DNA sequence itself but influence gene expression and it includes DNA methylation, histone modifications, and non-coding RNAs [13]. DNA methylation is the most widely studied epigenetic mark, which is essential for mammalian development, crucial for the establishment and maintenance of cell identity, and it affects gene expression by regulating promoters and distal regulatory elements, such as enhancers and insulators [14,15,16]. It occurs most often on a cytosine leading a guanine, which are referred to as CpG dinucleotides. They are globally underrepresented in the genome, except in CpG islands. CpG islands are CpG-dense regions largely resistant to DNA methylation [17,18]. They are generally found at promoters of housekeeping and developmental genes, and are represented in 70% of the promoters, but can be also found in exons, introns and regulatory regions [19].

The dystrophia myotonica protein kinase (*DMPK)* gene (the gene carrying the disease causing CTG expansion in its 3′ untranslated region) and neighbouring genes (henceforth referred to as the *DMPK* locus) contain several CpG islands (CpGis). CpGi 374 has gained the most attention because this 3.5 kb island contains the expanded repeat. In addition, the CTG repeat is flanked by two CCTC-binding factor (CTCF) binding sites, named CTCF1 and CTCF2. Early studies suggested that the two CTCF binding sites together with the expanded repeat established an insulator element between the *DMPK* promoter and the six homeobox 5 (*SIX5*) enhancer, affecting chromatin dynamics [20]. Several studies have found aberrant DNA methylation profiles in CTCF1 and CTCF2 regions in DM1, but they do not reach consensus [20,21,22,23,24,25,26,27,28,29,30]. The effect of aberrant DNA methylation profiles in the CTCF1 region on clinical phenotype is still largely unknown. In a previous study, Yanovsky-Dagan and collaborators identified a new differentially methylated region in DM1-affected human embryonic stem cell lines, at the beginning of CpGi 374, 900 bp upstream of the CTG expansion, which corresponded to a SIX5 regulatory element within the *DMPK* coding sequence [30]. This finding showed the importance of DNA methylation changes inside the *DMPK* gene body (not only in the regions closest to the CTG expansion, containing the CTCF binding sites), and raises an interest for the study of the epigenetic state of the other CpG islands located at the *DMPK* gene in DM1.

The *DMPK* locus harbours three more CpG islands, one in the neighbouring myotonic dystrophy WD repeat containing (*DMWD)* gene, CpGi 74, and two in the *DMPK* gene, namely CpGi 43 and 36; however, nothing is known about the epigenetic state of these CpG islands in DM1. To date, only one publication has looked at the entire *DMPK* locus and this was solely done in control tissues and cell cultures [31]. Interestingly, they described muscle-associated DNA hyper- and hypomethylation in the *DMPK* gene neighbourhood, in regions containing CpGi 43 and CpGi 74, respectively. Furthermore, CpGi 43 overlaps with a proposed alternative *DMPK* promoter, which could be tissue-specific and epigenetically regulated. Therefore, the main goal of this study was to elucidate the DNA methylation profiles across the four CpG islands residing in the *DMPK* locus in distinct DM1 tissues and tissue-derived cells across the different clinical phenotypes. Our results showed a CTCF1 DNA methylation gradient in blood of the developmental cases and CTCF1 methylation correlated to disease severity and CTG expansion size. Methylated cases showed a higher chance of maternal transmission and CTCF1 methylation in the parent was associated with a contraction of the CTG expansion upon generational transmission. Notably, DM1 patient-derived cells preserved the DNA methylation profiles observed in tissues. Finally, our results showed a DM1 muscle-specific epigenetic landscape, with a loss of methylation at CpGi 43, a region containing an alternative *DMPK* promoter, accompanied by a gain of methylation in the CTCF1 region in muscle and muscle-derived cells. Altogether, our results offer novel insights into the epigenetic changes in DM1.

## 2. Materials and Methods

### 2.1. Patient Registry

This study was approved by the Ethics Committee of the University Hospital Germans Trias i Pujol and was performed in accordance with the Declaration of Helsinki for Human Research. Written informed consent was obtained for all participants. The study included 65 DM1 patients and 8 controls with no previous family history of neuromuscular disorders (recruited from the traumatology department in whom surgery was needed). DM1 diagnosis was confirmed or discarded with triplet primed-PCR in all the study participants. Clinical information of DM1 patients was obtained from the medical records and updated in the last visit by neurologists. Patients were subdivided into five different categories based on age of onset: congenital = first year of life, childhood = 1–10 years, juvenile = 11–20 years, adult = 21–40 years, late-onset > 40 years. Additionally, a group of asymptomatic patients was added. For three patients the exact year of age of onset could not be determined, but based on the clinical information all three were classified as adults. Clinical information included family history, and details on the last ophthalmological, cardiological and respiratory examination by the corresponding specialists, including the electrocardiograms, echocardiograms and spirometry tests performed in the last year. A full neurological work-up was performed by neurologists, including the presence of myotonia, ptosis, axial and facial weakness, muscle strength with the manual Medical Research Council (MRC) scale, and muscle impairment by the Muscular Impairment Rating scale (MIRS). In addition, the presence of cataracts, alopecia, intestinal problems, and sleep disturbances were catalogued and the functional status and degree of disability were evaluated using the DM1-Activ questionnaire and modified Rankin Scale (mRS), respectively.

### 2.2. Tissue and Cell Culture

A total of three different samples from patients and controls were obtained: blood, muscle biopsy, and skin biopsy. Blood was obtained from all patients and lymphoblastoids were isolated when possible. From a subset of patients and controls, an additional muscle (biceps brachialis or vastus lateralis) and skin biopsy were taken, of which muscle and skin-derived cell cultures were obtained. Of note, to increase the number of biopsies, a subset of biopsies for which no blood was available were included. They included three extra patients and two controls. All samples were obtained at the same time and processed as described previously by Ballester-López and collaborators [32]. Cell isolation and subsequent culturing was performed as previously described by Koehorst and collaborators [33].

### 2.3. DNA Isolation

Genomic DNA was isolated from peripheral blood by the use of the QIAamp DNA mini kit (Qiagen, Hilden, Germany), the PureLink genomic DNA mini kit (Thermo Fisher Scientific, Waltham, MA, USA) or a simple salting procedure, as previously described by Miller and collaborators [34]. Genomic DNA from muscle and skin tissue was extracted as previously described by Ballester-López and collaborators [32].

### 2.4. CTG Expansion Size Analysis

To estimate the length of the expanded progenitor allele, a specific long PCR with digested DNA, followed by a Southern Blot was carried out. First, 250 ng DNA was digested with EcoR I (New England Biolabs, Ipswich, MA, USA), according to the manufacturer’s protocol. Then, 750 pg of digested DNA was used in the subsequent PCR, with the Expand Long Template PCR System (Roche, Basel, Switzerland) and primers DM-C and DM-DR (Table S1), according to the manufacturer’s guidelines, supplemented with 2% DMSO. The following thermocycler conditions were used: initial 3 min at 96 °C, followed by 28 cycles of 15 s 96 °C, 45 s 63.5 °C, 5 min 68 °C, and a final extension step of 1 min 63.5 °C and 7 min 68 °C. DNA fragments were resolved by electrophoresis on a 1% agarose gel. The gel was run for an initial 10 min at 200 V, followed by ±19 h at 27 V and blotted onto a positively charged nylon membrane (Roche, Basel, Switzerland). The membrane was hybridized with a digoxigenin labelled seven CAG LNA probe overnight and detected by chemiluminescence using the anti-Dig-CDP-Star system (Roche, Basel, Switzerland). Two CTG expansion sizes were estimated through comparison against the molecular weight ladder using GelAnalyzer 19.1 software (www.gelanalyzer.com, by Istvan Lazar Jr. and Istvan Lazar Sr., accessed on 16 February 2022). The CTG size of the progenitor (ePAL), which is the lowest range of band thought to originate from the transmitting parent, and the modal allele, which shows the densest collection of CTG sizes and thought to be the most representative size for the patient at that specific time.

### 2.5. Bisulphite Treatment and Sanger Sequencing of Four CpG Islands

The methylation status of four annotated CpG islands (CpGi) in the *DMPK* locus, divided into five individual areas, was studied by using bisulphite treatment and subsequent Sanger sequencing. For CpGi 74, CpGi 43 and CpGi 36, 19, 15 and 17 CpGs were studied respectively (Figure S1A–C for detailed location). In the CPGi 374, two separate regions were studied, namely CTCF1 and CTCF2, which surround the CTG expansion and each hold a CTCF binding site. For CTCF1, 25 CpGs were studied and for CTCF2, 11 CpGs were studied (Figure S1D,E for detailed information).

A total of 200–400 ng of DNA was bisulphite treated using the EZ DNA Methylation Gold kit (Zymo Research, Irvine, CA, USA), according to the manufacturer’s guidelines. Bisulphite-treated DNA was amplified using nested and hemi-nested PCR for the CTCF1 and CTCF2 region, located in the CpGi 374 surrounding the CTG expansion, previously described by Barbé and collaborators [21], with some minor modifications. Here we used the TaKaRa Taq DNA polymerase (TaKaRa Bio Inc., Kioto, Japan) on a Mastercycler nexus X2 thermocycler, primer combinations and thermocycler settings are listed in Table S1. Additionally, three regions further upstream of the CTG repeat were analysed, namely the CpGi 36, CpGi 43 and CpGi 74 regions, in a similar fashion, using different primer combinations and thermocycler settings (Table S1).

Amplicons were purified using Illustra™ ExoProStar 1-Step (Merck, Darmstadt, Germany), according to the manufacturer’s protocol. This was followed by sequencing using the BigDye Terminator v3.1 cycle sequencing kit (Thermo Fisher Scientific, Waltham, MA, USA), following the manufacturer’s guidelines. Afterwards, amplicons were run on an ABI Prism 3130 Genetic Analyzer (Applied Biosystems, Waltham, MA, USA) and analysed using Chromas software version 2.6.6 or FinchTV software version 1.5.0, as detailed in Carrió et al. 2016 [35]. Sodium bisulphite sequencing data were represented with the Methylation Plotter web tool [36]. A CpG island was considered methylated when the majority of CpGs studied for that particular island (>50%) showed ≥10% methylation.

### 2.6. Statistical Analysis

Dichotomous variable CTCF1 methylation status, the occurrence of abnormal methylation upstream of the repeat, was modelled as a dependent variable using a logistic regression model, against the independent variable ePAL and modal allele. The program used was SPSS 28.0.0.0 and the significance level was set at 0.05.

## 3. Results

### 3.1. A Study Cohort Encompassing All Clinical Subtypes of DM1

For this study, DM1 patients from six different subcategories were included. The first five categories are the different established clinical phenotypes: congenital, childhood, juvenile, adult and late-onset. The sixth category is a special subset of patients, which are known to carry the CTG expansion, but are as of yet asymptomatic. Congenital (*n* = 6), childhood (*n* = 6) and juvenile (*n* = 23) will also be referred to as the developmental cases, whereas adult (*n* = 22), late onset (*n* = 6) and asymptomatic (*n* = 2) will be referred to as the non-developmental cases. Age of onset ranged from first year of life until 67 years, with a mean of seven years for childhood, 15 years for juvenile, 31 years for adult and 52 years for late-onset. The presence of seven families was identified in this cohort. In 59 out of 65 patients, the CTG expansion could be sized, ranging from 115 to 1011 CTG repeats. Detailed information on the clinical phenotypes can be found in Table 2.

### 3.2. DNA Methylation Profiles across the DMPK Locus in Blood

**Figure 1:**
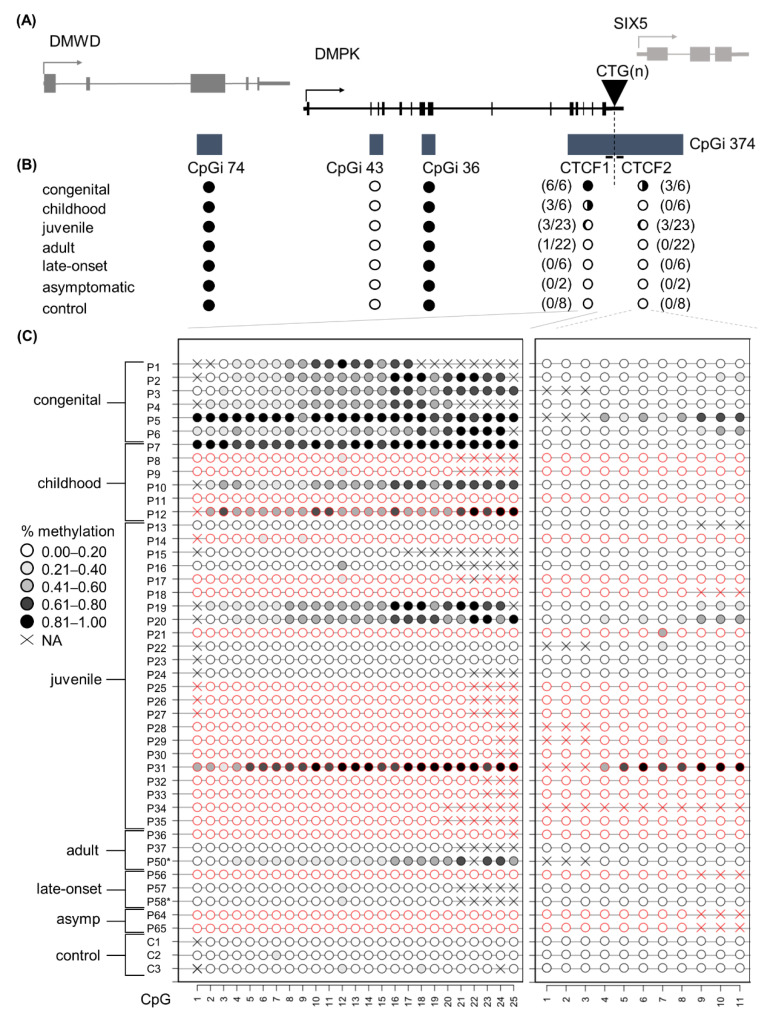
DNA methylation profile at the *DMPK* locus in DM1 blood samples representing all clinical subtypes. (**A**) Schematic representation of the four CpGis residing in the *DMPK* locus and neighboring genes. CpGi 374 is divided into the CTCF1 and CTCF2 region, harboring the CTCF binding sites and encompassing the CTG expansion. (**B**) Summary of the methylation profiles of the five CpG regions across the *DMPK* locus in the studied clinical subtypes, in which black indicates the degree of methylation. Congenital *n* = 6, Childhood *n* = 6, Juvenile *n* = 23, Adult *n* = 22, Late onset *n* = 6, asymptomatic *n* = 2, Controls *n* = 8. (**C**) Detailed DNA methylation profiles of the clinical subtypes in the CTCF1 and CTCF 2 regions. Each circle represents a CpG dinucleotide. The colour gradient represents the level of methylation indicated in the legend assessed by sodium bisulphite sequencing. Red indicates paternal inheritance. Black indicates maternal inheritance. * means unknown inheritance. For the non-developmental cases, only a representative subset of three samples is displayed. Detailed methylation profiles of all patients in all categories can be found in the supplemental tables. Abbreviations: DMWD = dystrophia myotonica WD repeat-containing gene, *DMPK* = myotonic dystrophy protein kinase gene, SIX5 = six homeobox 5 gene, CTG(n) = the CTG expansion, CpGi = CpG island, Asymp = asymptomatic, C = control, P = DM1 patient.

This study aimed to elucidate the DNA methylation profiles across the four CpG islands residing in the *DMPK* locus in distinct DM1 patient samples and derived primary cell cultures. The four CpGis were divided into five distinct regions: CpGi 74, CpGi 43, CpGi 36, CTCF1 and CTCF2. The latter two reside in the same CpG island and refer to the two regions that contain a CTCF binding site and also encompass the CTG expansion (Figure 1A). In blood samples, for the first three CpGis, no differences in DNA methylation levels across the six phenotypes and the controls were observed. CpGi 74 and CpGi 36 showed high levels of methylation (90–100%) across the 19 and 17 CpGs studied, respectively (Figure 1B and Tables S2 and S4), meanwhile CpGi 43 showed no methylation across the 15 CpG sites studied (Figure 1B and Table S3). For the CTCF1 region upstream of the CTG repeat, 25 CpG sites were studied and increased levels of methylation were observed almost exclusively in the developmental cases, with 100% of the congenital cases, 50% of the childhood cases and 13% of the juvenile cases (Figure 1C and Table S5). No methylation was found in the non-developmental cases and the controls, except for one adult case out of 30 (P50). The two CpGs (CpG 18 and 19) that reside inside the proposed CTCF1 binding site were both highly methylated in the methylated cases. Regarding the CTCF2 region found downstream of the CTG repeat, eleven CTG sites were studied and fifty percent of the CTCF1 methylated cases also showed methylation in the CTCF2 region (Figure 1C and Table S6). No CTCF2 methylation was found in cases that were not methylated in CTCF1. CpG 5 resides in the CTCF2 binding site and it was partially methylated in the found methylated cases. Interestingly, for one of the congenital cases (P1), we obtained another blood sample from a five-year follow-up. The methylation pattern that this patient showed in the CTCF1 region was preserved after five years, whereas CTCF2 remained unmethylated (Tables S5 and S6, annotated as P1 and P1–2).

### 3.3. Aberrant DNA Methylation Profiles of CTCF1 Associated with Higher Disease Severity in Childhood Cases

Since the methylation profiles showed exclusive methylation in the CTCF1 region of the developmental cases, we reviewed their clinical phenotypes in-depth to see whether this aberrant methylation profile is associated with a differential disease severity. The congenital cases all showed high methylation and no clinical phenotype distinction based on DNA methylation status could be made (detailed clinical information in Table S7). Our focus was therefore on the childhood and juvenile cases. In the group of childhood-onset DM1, three out of six patients showed methylation (detailed clinical information in Table 3). One case with methylation was female; all the other childhood cases were male. Age of onset was on average 6.83 years old, but the age at sampling was a few decades later with a mean of 41.83 years.

At the time of revision, all patients showed muscle weakness and myotonia, but the muscular symptoms seemed to be more significant in the methylated group. Two out of three of the methylated patients experienced cramps and myalgia, while just one non-methylated patient suffered this symptom. All six patients had the characteristic facial dysmorphia and ptosis. The ptosis seemed to be more pronounced in the methylated patients: two of the patients had a severe ptosis covering the pupil and the last one had a moderate ptosis covering part of the pupil. In the non-methylated group, just one patient had a moderate ptosis, while the other two had a mild ptosis. Facial weakness was also present in all childhood-onset-DM1 patients, being severe in 1/3 and moderate in 2/3 of the methylated patients; whereas the non-methylated group showed only mild (two patients) and moderate facial weakness (one patient). Dysarthria was present in all patients, but to a higher degree in methylated patients, where it ranged from moderate to severe, compared to mild to moderate in non-methylated patients. Axial and limb weakness was also more pronounced in the methylated versus the non-methylated group. In the methylated cases, 2/3 had a severe limb weakness (MRC scale of 1–2): one of the patients had a proximal and distal weakness, requiring a wheelchair and the other patient showed a proximal and distal weakness pattern needing just a cane for walking. The last one of the methylated childhood cases had a mild distal weakness (MRC scale 3–4). In the non-methylated group, all of the patients showed a mild weakness (MRC of 3–4), two of the patients with a distal pattern and one patient with proximal/distal weakness.

Cardiac manifestations were more severe in the methylated group, as 2/3 patients had a pacemaker, whereas in the case of the non-methylated group only mild changes in electrocardiogram were seen, such as a mild first grade AV block in 2/3 cases. Two of the methylated patients and one of the non-methylated patients used ventilatory support. Cognitive manifestations were noticed in all methylated childhood DM1 patients: two of the patients had severe cognitive delay, while moderate learning difficulty was observed in the third patient. In the non-methylated group, just one patient had learning difficulties, while the others showed no mental disabilities. All methylated patients experienced hypersomnolence and none of the non-methylated patients suffered this symptom. Intestinal rhythm dysfunction was found exclusively in the methylated patients, while alopecia was found in the non-methylated patients only.

At the time of revision, all patients had some degree of dependence in the mRS. In the methylated group, 2/3 needed help in the basic activities of daily life but did not require continuous supervision (mRS 4), while the other one required help for instrumental activities (mRS 3). The average score in the DM1-ACTIV scale was 12.33. In the non-methylated group, we found one patient with mRS 4, another one with mRS 3 and the last one had a milder dependence (mRS 2) and the average score in the DM1-ACTIV scale was 28.33, which means they were better at performing daily and social activities. Considering all this data as a whole, there seemed to be a more severe muscular, cardiac, and cognitive manifestation of the disease in the methylated childhood cases.

Upon revision of the juvenile cases, no such differences in muscular, cardiac and cognitive manifestation could be found between the methylated (*n* = 3) and non-methylated group (*n* = 20). Although, this might be due to the low number of cases, and analysis of a larger cohort of this DM1 subcategory would be needed to address the impact of CTCF1 methylation on this clinical subtype (detailed information in Table S8).

### 3.4. A Higher Chance of Methylation in CTCF1 with Increasing CTG Expansion Size

As mentioned before, methylation of the CTCF1 region is almost exclusively found in the developmental subtypes. These subtypes are associated with a higher disease severity and overall greater CTG expansion sizes. To see whether methylation status in DM1 patients is associated with the CTG expansion size, we performed a logistic regression on the entire cohort using the ePAL and modal allele. This revealed a positive association between ePAL and methylation status of the CTCF1 region in DM1 patients (Table S9, model 1, *p* = 0.004) and between the modal allele and the methylation status (Table S9, model 2, *p* = 0.001), which suggests that the larger the ePAL/modal, the more likely methylation at the CTCF1 regions is going to occur.

### 3.5. Increased, but Not Exclusive, Maternal Transmission in CTCF1 Methylated Cases

It has been reported that the methylation observed in DM1 cases is associated with maternal transmission. We therefore decided to evaluate, where possible, the transmission in this DM1 cohort (Figure 1C). We found that all congenital cases, which were all hypermethylated, were maternally transmitted. In both childhood and juvenile cases, two out of the three methylated cases in each category were maternally transmitted. For the childhood subcategory as a whole, a total of two maternal transmissions were registered, meaning that all maternally transmitted cases reside in the methylated group. However, for the juvenile subcategory, a total of seven patients were maternally transmitted, of which only two reside in the methylated group. Taken together, we could corroborate a higher chance of maternal transmission when methylated, but notably there were patients that were methylated and paternally transmitted.

### 3.6. Methylation Status Is Not Inheritable and Associated with the Transmission of CTG Repeat Contractions

**Figure 2:**
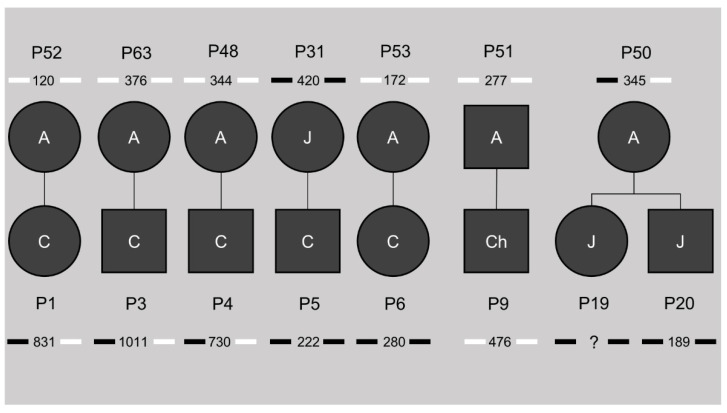
Pedigrees of the known families in our study cohort. The number beneath the patient identification code indicates the ePAL (estimated progenitor allele size), with the bars next to it indicating methylation status of CTCF1 (left) and CTCF2 (right). Black indicates methylation, white no methylation. A = adult, J = juvenile, Ch = childhood, C = congenital. ? = unknown CTG expansion size.

This study cohort included seven families, giving us the opportunity to study the inheritance of the differential DNA methylation profiles shown in CTCF1 and CTCF2 (pedigrees in Figure 2). Of five out of six congenital cases, the mother was included in the study cohort as well. Only one of the mothers showed methylation at both the CTCF1 and CTCF2 region, whereas the other four were unmethylated. This methylated mother belonged to the juvenile subtype. For the childhood subset, only one family could be studied, where both the childhood case and the adult-onset father were unmethylated. For the juvenile subset, one family consisting of two siblings with both juvenile onsets could be studied. These two siblings were both methylated at the CTCF1 and CTCF2 regions and interestingly the mother was the only adult methylated case in our cohort, showing only CTCF1 methylation. Of note, when reviewing the CTG expansion sizes, the unmethylated mothers passed on an expansion of the CTG repeat, whereas the methylated mothers transmitted a contraction of the CTG repeat.

### 3.7. DNA Methylation Profiles Are Preserved in Blood-Derived Cells

**Figure 3:**
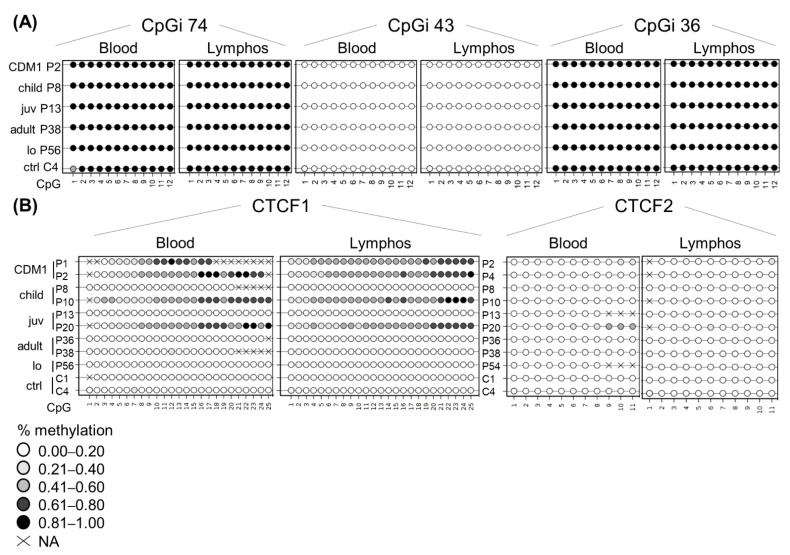
DNA methylation profile is preserved at the *DMPK* locus in patient-derived lymphoblastoids. Comparison of the DNA methylation profile between blood and lymphoblastoid cell lines of the same individual across the five annotated CpG regions: (**A**) CpGi 74, 43 and 36. (**B**) CTCF1 and CTCF2. Each circle represents a CpG dinucleotide. The colour gradient represents the level of methylation indicated in the legend assessed by sodium bisulphite sequencing. Abbreviations: C = control, P = DM1 patient; CDM1 = congenital, child = childhood, juv = juvenile, lo = late-onset, ctrl = controls, lymphos = lymphoblastoids.

To see whether lymphoblastoids preserve the epigenetic landscape and can be used as a faithful DM1 cellular model, we decided to study whether the DNA methylation profiles are similar between blood and the blood-derived lymphoblastoids (Figure 3). CpGi 74, CpGi 43 and CpGi 36 showed no differences between blood and lymphoblastoids across all clinical subtypes (Figure 3A and Tables S10–S12). Both CpGi 74 and CpGi 36 remained completely highly methylated regions, whereas CpGi 43 was totally unmethylated. For CTCF1, the pattern observed in blood, a gradient of methylation in the developmental cases was preserved in all the studied lymphoblastoids (Figure 3B), except for P7, which showed methylation in blood, but not in lymphoblastoid cells in CTCF1 (Table S13). Regarding CTCF2, the patients that displayed methylation of the CTCF2 region in blood, preserved their methylation status in the studied lymphoblastoids (Figure 3B and Table S14). However, two patients that were not methylated in blood for CTCF2 (P4 and P10) showed low-grade methylation in lymphoblastoids (~10% methylation) (Table S14).

### 3.8. DM1 Is Associated with Hypomethylation of CpGi 43 in Muscle Tissue and Muscle-Derived Cells

**Figure 4:**
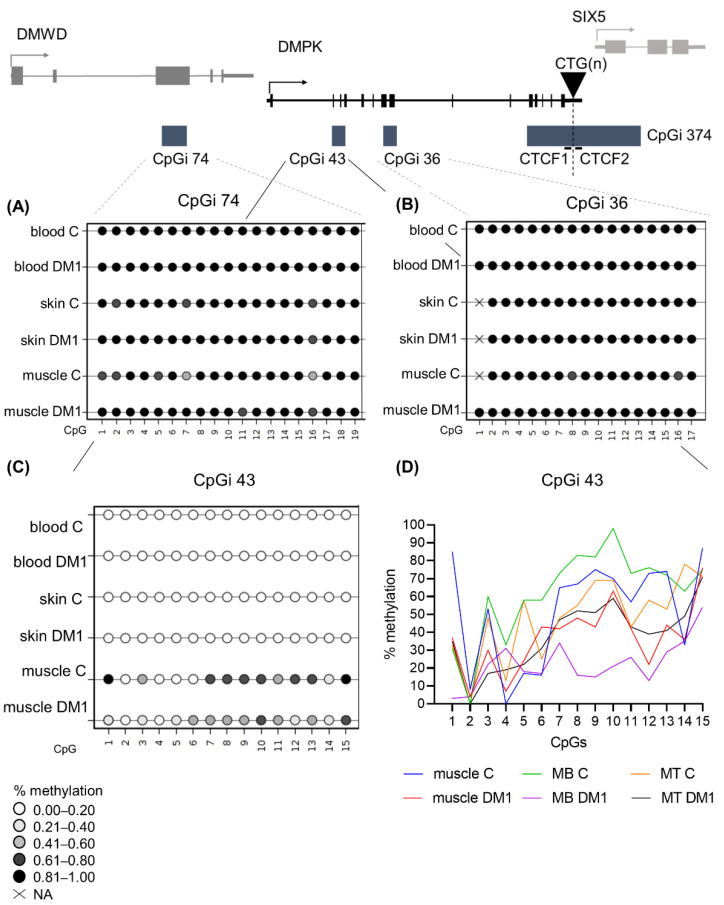
DNA methylation profile at CpGi 43 showed specific hypomethylation in DM1 muscle tissue and muscle-derived cells. (**A**–**C**) Averages of DNA methylation profiles across the three studied tissues, blood, skin and muscle, in patients versus controls for CpGi 74 (**A**), for CpGi 36 (**B**) and CpGi 43 (**C**). Included patients are from the juvenile, adult and late onset clinical category (*n* = 5 for blood, *n* = 3 for skin, *n* = 7 for muscle and *n* = 4 for controls). Each circle represents a CpG dinucleotide. The colour gradient represents the level of methylation indicated in the legend assessed by sodium bisulphite sequencing. (**D**) Representation of the DNA methylation profiles of muscle and muscle-derived cells (myoblasts and myotubes) in CpGi 43 assessed by bisulphite sequencing.

Next, we wanted to study the DNA methylation profiles of the five regions in tissues other than blood to address whether tissue-specific epigenetics at the *DMPK* locus exists in DM1. For this, we acquired a muscle and skin biopsy from a subset of patients (juvenile, adult and late-onset origin). From these biopsies, cells were isolated to assess whether cellular models accurately reflect the origin tissue in terms of DNA methylation status. In CpGi 74 and CpGi 36, high DNA methylation levels were found in skin and muscle of both DM1 patients and controls, similar to what was found in blood (Figure 4A,B). Additionally, the tissue-derived cells of both muscle and skin preserved the observed methylation (Tables S15–S18). Regarding CpGi 43, skin samples and skin-derived cells showed no methylation in the DM1 patients and controls, similar to the observations in blood, with the exception of one patient and one control in skin fibroblasts (Figure 4C, Table S19). Interestingly, a distinct DNA methylation pattern was observed in muscle tissue, with overall high methylation in control samples, which was much lower in DM1 muscles, showing reductions as high as 70% in some CpGs (Figure 4C,D and Table S20). This muscle-specific DNA methylation profile was preserved in both control- and patient-derived myoblasts and myotubes (Figure 4D and Table S20). Interestingly, DM1 myoblasts or myogenic precursor cells showed the biggest DNA demethylation compared to controls.

### 3.9. DM1 Is Associated with Hypermethylation in CTCF1 in Muscle Tissue and Muscle Derived Cells

**Figure 5:**
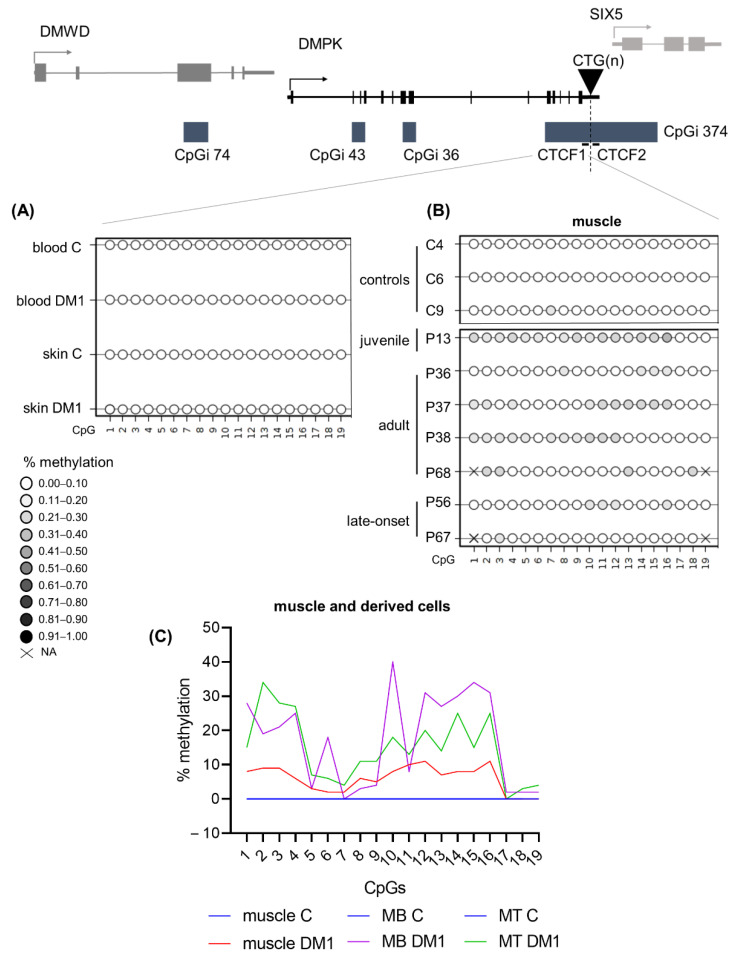
DNA methylation profiles at the CTCF1 regions were tissue-specific and increased in DM1 muscle samples. (**A**) Averages of DNA methylation profiles across blood and skin in patients versus controls for CTCF1. Included patients are from the juvenile, adult and late onset clinical category (*n* = 5 for blood, *n* = 3 for skin and *n* = 4 for controls). (**B**) Overview of the muscle biopsy DNA methylation profiles of DM1 patients and controls. Each circle represents a CpG dinucleotide. The color gradient represents the level of methylation indicated in the legend assessed by sodium bisulfite sequencing. (**C**) Representation of the DNA methylation profiles of muscle and muscle-derived cells (myoblasts and myotubes) in CTCF1. For CTCF1 all control muscle cells are at zero and have been given the same color to aid visualization.

The CTCF1 region showed similar DNA methylation profiles in skin and skin-derived cells compared to blood (Figure 5A and Table S21). However, one DM1 skin fibroblast sample showed low-grade methylation (average of around 10%), but none in controls (Table S21). Due to difficulties with the sequencing analysis, only in one patient could both the skin and the skin-derived fibroblasts be analysed. For the other samples, we analysed DM1 skin fibroblasts that were not derived from the analysed skin biopsies; therefore, we cannot rule out the possibility that this sample already showed a partial methylation, especially since it is a juvenile sample. Interestingly, six out of seven DM1 muscle biopsies showed hypermethylation compared to controls, with half of the samples showing a gain of methylation in at least 50% of analysed CpGs (Figure 5B and Table S22). Notably, the highest methylation average was found in the youngest biopsy, belonging to a juvenile case. Of these biopsies, we have the CTG expansion size available in muscle and blood (Table S23). However, CTG expansion size could not be linked to the degree of methylation. Surprisingly, the two CpGs residing in the CTCF1 binding site (CpG 18 and 19) were not methylated in these biopsies, with the exception of CpG 18 in the biopsy of P68. Total unmethylation in all CpGs was observed in the control biopsies. This methylation profile was maintained in the muscle-derived cells (Figure 5C and Table S22). Of note, the differences in myogenic precursor cells between patients and controls were the highest (Figure 5C). The last analysed region, CTCF2, showed no methylation in the tissues and tissue-derived cells studied from the patients of which we have the biopsies (Tables S24 and S25), suggesting that muscle-specific hypermethylation only happens in the CTCF1 region.

## 4. Discussion

**Figure 6:**
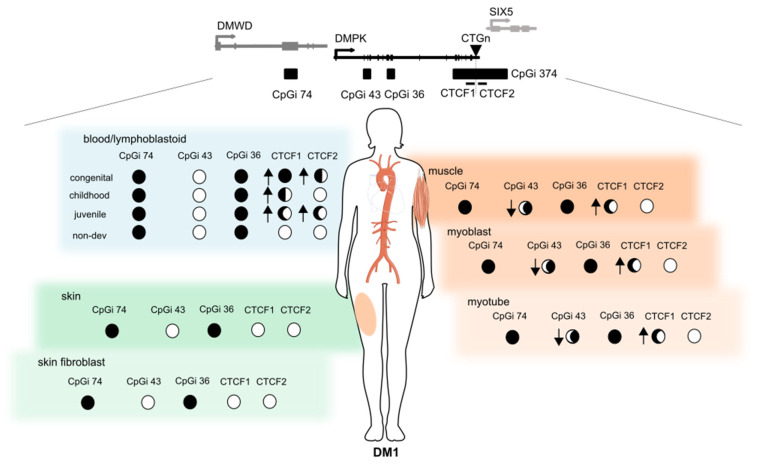
Summary of the epigenetic landscape of the *DMPK* locus in DM1. An overview is given of the methylation status across the *DMPK* locus in muscle, skin and blood and their derived cells in DM1 patients. The circle underneath each CpG island represents its methylation status, ranging from fully methylated (black) to completely unmethylated (white) in DM1 patients. The arrows indicate the changes compared to controls, where an upward arrow means increased methylation in DM1 compared to controls. This figure was partially made using Servier Medical Art (smart.servier.com, accessed on 31 March 2022).

The overall goal of this study was to investigate the contribution of epigenetics to DM1 pathology, by analysing for the first time the DNA methylation profiles across the four CpG islands residing in the *DMPK* locus in several tissues and tissue-derived cells in all clinical subtypes of DM1. Our results showed a distinct DNA methylation profile across DM1 tissues and uncovered a novel and dual epigenetic signature in DM1 muscle samples, involving a gain of DNA methylation in the flanking region of the CTG expansion accompanied by specific DNA demethylation in the *DMPK* gene body (Figure 6).

Previously, it was reported that in leukocytes derived from control individuals, CpGi 36 and 74 (located in the *DMPK* and *DMWD* gene bodies, respectively) were highly methylated, while CpGi 43 (located in *DMPK* gene body and overlapping with a proposed alternative promoter) was unmethylated [31]. Our results showed that in DM1 blood samples, the DNA methylation profile of CpGi 36, 43, and 74 does not change in the distinct DM1 subtypes. Conversely, the CTCF1 and CTCF2 regions did show a change in DNA methylation levels in DM1 compared to controls. Developmental cases showed an upward gradient of methylation with increasing severity of the disease and decreasing age of onset. No methylation was observed in the non-developmental cases, except for a single adult case. Additionally, fifty percent of the CTCF1 methylated cases also showed methylation in the CTCF2 region, but interestingly, CTCF2 methylation without CTCF1 methylation was not observed in any case. This may indicate that the beginning of the aberrant DNA methylation is not random and it spreads beyond the CTG repeat only in certain cases/conditions. The mechanism behind the high methylation levels observed in the CTCF1 region and why the mechanism seems to be biased towards developmental cases are still relevant questions that need addressing in the DM1 field.

The current studies on DNA methylation profiles in the two CTCF binding regions are controversial. Nevertheless, several studies have shown a similar trend in the levels of CTCF1 methylation as we observed in our developmental cases [21,23,27,28]. Regarding the CTCF2 region, our results corroborate previous findings that CTCF2 is less methylated than the CTCF1 region [21,27,28]. Several studies have focused solely on adult cases, and have found methylation among these non-developmental cases, contradictory to our findings [24,25,37], although the methylation levels were very small in these studies. The differences found between the published works could be due to the different techniques used to assess DNA methylation levels, the different criteria used to decide what is considered methylated, and the difficulties to establish the age of onset in DM1. To better identify the DNA methylation differences in DM1 samples, a general consensus in DM1 DNA methylation studies would be needed. In our cohort we could see whether the methylation status changes over time, since we had the five-year follow-up of one of the congenital cases, where we found that the methylation status was stable, which is in concordance with previous studies [24,25,26].

Few studies have addressed the link between clinical phenotype and DNA methylation profiles. Due to the extensive clinical data obtained from this cohort, we had the opportunity to assess whether DNA methylation status was associated with clinical phenotype. Methylated childhood cases showed more severe muscular, cardiac, and cognitive manifestations of the disease. This type of phenotypic association in this particular disease subtype has not been made previously and the few available studies on clinical phenotype correlations are from adults. Légaré and collaborators have shown that methylation status is linked to muscular and respiratory profiles in adults [37] and Breton found a correlation between hypermethylation and a decline in cognitive function [24]. However, it has been stipulated that methylation seems to be associated with the more severe forms of the disease, as methylation is found overwhelmingly in CDM1 cases [21,27,28,29]. Our study supports this notion and adds the novel finding that it is also linked to the more severe cases of childhood DM1. Caution must be taken, however, as our sample size was quite low and further studies are needed to confirm this association.

Disease severity and age of onset have been previously linked to the CTG expansion size, where the more severe disease forms and earlier age of onset correlate with the greater CTG expansion sizes [38,39]. We found a positive association between two CTG size predictors and methylation status of CTCF1. This suggests that the larger the CTG expansion size, the higher the likelihood of CTCF1 methylation. This association has been found previously by several studies, both for ePAL [27] and modal allele [25,28,37]. However, not all studies were able to find such an association between CTG expansion size and the methylation status [21,23].

Our study cohort included several families, giving us the opportunity to study inheritance of the DNA methylation profiles. We found the DNA methylation status of our patients to be not inheritable, as several unmethylated mothers gave birth to methylated cases. This is in accordance with previous studies [21,27]. Interestingly, we observed that the offspring of methylated mothers carried contractions of the CTG expansion, while the offspring of unmethylated mothers carried expansions of the repeat. This suggests that although the bigger CTG expansion sizes are associated with methylation, when a methylated parent passes on the methylation status, it coincides with the transference of a smaller CTG expansion. Some authors have evaluated the effect of DNA methylation on the stability of the CTG expansion repeat [40]. When using bacterial and primate cellular models of 83 to 100 CTG repeat expansions, DNA methylation was found to be associated with a stabilization of the repeat size. However, caution must be taken with these observations, as the sample size in their study and our study was low and further studies are needed to further elucidate this observation.

We found an increased maternal transmission rate in CTCF1 methylated cases. However, this was not absolute. Barbé and collaborators have suggested the presence of a parent-of-origin effect, where DNA methylation may account for the maternal bias for CDM1 transmission, the larger maternal CTG expansions, age of onset, and clinical phenotype [21]. The hypothesis is based on the potentially reduced survival of spermatozoa due to the hypermethylation of the CTCF1 region, disrupting the insulator element and decreasing levels of *SIX5*, which is essential for spermatozoa survival. Although we do see a similar trend, as all our CDM1 cases are both maternally transmitted and methylated, parental inheritance might not be a good predictor for methylation status or vice versa for the other clinical phenotypes. This is strengthened by the observation made by Morales and collaborators, where a large family showed several paternally transmitted methylated cases [27]. Furthermore, a very recent study in DM1 spermatozoa found that methylation did not affect sperm viability and these spermatozoa were compatible with “in vitro” fertilization [41], which goes against the hypothesis that reduced survival is associated with methylated spermatozoa, preventing the transmission of CDM1 [3], and therefore, other explanations for this maternal bias should be explored.

Buckley and collaborators extensively studied the epigenetics in the *DMPK* locus in several control tissues and cell types. Interestingly, they reported the existence of a *DMPK* alternative promoter (also referred to as the downstream promoter, which overlaps with CpGi 43), as well as cell type-dependent differences in promoter usage, according to epigenetic features [31]. The data presented by Buckley and collaborators showed a predominant use of the canonical upstream promotor in skeletal muscle and myogenic cells from control individuals, by RNA-CAGE (cap analysis gene expression) data, supported by complete hypomethylation of this promoter and hypermethylation of the alternative downstream promoter [31]. Conversely, in blood, a predominant usage of the alternative *DMPK* promoter (in CpGi 43) was suggested, by showing its hypomethylation together with hypermethylation of the canonical promoter, strong binding of CTCF and high levels of H3K4me3 in control leukocytes [31]. Interestingly, our study of DM1 muscle samples revealed a novel epigenetic change by specific demethylation of this alternative promoter located at CpGi 43, in skeletal muscle tissue and muscle-derived cells. This could potentially alter chromatin conformation and result in a shift in the promoter usage from the strongest/canonical one to the weak/alternative promoter, decreasing *DMPK* expression levels in DM1 myogenic samples.

Additionally, this DM1 tissue-dependent demethylation was accompanied by a gain of methylation in the CTCF1, but not the CTCF2 region. Previous studies showed that CTCF, a transcription factor that can function as an insulator, binds strongly to CTCF1, but not CTCF2, in a methylation-dependent manner [20]. Importantly, the hypermethylation of the CTCF regions could inhibit CTCF-binding and disrupt the insulator element formed by the CTG expansion and the two CTCF-binding sites, affecting *DMPK* and *SIX5* expression. The loss of the insulator activity by DNA methylation would allow the interaction of the *SIX5* enhancer with the *DMPK* promoter, increasing *DMPK* expression, while reducing *SIX5* expression [20]. Notably, our results showed that in blood samples and derived-leukocytes, the CpGs located inside the CTCF1 and CTCF2 binding sites were hypermethylated in all methylated cases (almost exclusively developmental cases), while in muscle samples, these remained unmethylated (almost exclusively non-developmental cases). This may imply that although there is a disease-specific gain of methylation for the CTCF1 region in muscle, the CTCF binding site is not disrupted, allowing CTCF binding. However, this hypermethylation might affect other chromatin interactions, in turn affecting gene expression. The analysis of publicly available ChIP-seq data of histone post translational modifications (H3K4me3, H3K4me1 and H3K27ac), done by Buckley and collaborators at *DMPK* and neighbouring genes, showed that CTCF regions and intragenic regions of *DMWD* and radial spoke head 6 homolog A genes (*RSPH6A*), located next to the *DMPK* gene, displays enhancer chromatin features in control muscle cells [31]. This is interesting since *DMPK* lies in the middle of a chromosomal domain with three genes preferentially expressed in testis, indicating that its expression, mainly in skeletal muscle and heart, has to be tightly regulated. Additionally, Brouwer and collaborators showed an increase in the H3K9me3 chromatin repressive mark, together with gain of DNA methylation, in the CTCF1 region (and to a lesser extent in CTCF2) in DM1 mice hearts, which correlates with decreased *DMPK* expression [22]. To address whether DNA methylation changes in the CTCF1 region in DM1 skeletal muscles may alter chromatin interactions between the *DMPK* promoter and these potential myogenic enhancers, further experiments are needed.

Finally, this study addressed for the first time the DNA methylation status of patient-derived DM1 cells. The availability of several DM1 tissues and their corresponding tissue-derived cells gave us the opportunity to determine that most cellular models maintained the DNA methylation state observed in the original tissue. However, in some cases, we observed a slight gain of methylation in cultured cells (e.g., some DM1 lymphoblastoids, skin fibroblasts and myoblast/myotubes) versus the corresponding tissues. This can be explained by the observation that cellular models, especially immortalized cell lines or primary cell cultures that have been in culture for a substantial amount of time, can increase DNA methylation levels [13,42,43] and/or because of the purity of cell cultures compared to tissues containing distinct cell types. Overall, our results showed that the DM1 patient-derived cells preserve the genetic and epigenetic features, which makes them excellent models to study DM1 pathology.

## 5. Conclusions

In conclusion, our results showed a distinct DNA methylation profile across DM1 tissues and uncovered a novel, dual epigenetic signature involving a gain of DNA methylation in the flanking region of the CTG expansion, accompanied by specific DNA demethylation in the *DMPK* gene body of DM1 muscle samples, which provided novel insights into the epigenetic changes occurring in DM1.

## Figures and Tables

**Table 1:** Clinical manifestations of the different DM1 subcategories.

Clinical Subtype	Age of Onset	Main Symptoms
Congenital	<1 year	Poor fetal movement Hypotonia Feeding difficulties Clubfoot deformities Respiratory failure Learning disability Cardiorespiratory complications
Childhood	1–10 years	Cognitive and learning disabilities Facial weakness Myotonia Conduction defects
Juvenile	11–20 years	Skeletal muscle weakness Myotonia Cognitive and learning disabilities Conduction defects
Adult	21–40 years	Progressive muscle weakness Myotonia Early-onset cataracts Conduction defects Endocrine dysfunction
Late-onset	>40 years	Low-grade muscle weakness Early-onset cataracts alopecia

**Table 2:** Clinical characteristics cohort.

Clinical Subtype	*n* Patients	Age of Onset (Years)	Age at Sampling (Years)	Inheritance Maternal	Gender (Male)	ePAL (CTGs)	Myotonia	Biceps MRC Scale	MIRS	Cardiac Involvement	NVM	Cataracts	mRS
Congenital	6	At birth	12.83 ± 5.43	(6/6)	(4/6)	610 (222–1011)	(2/6)	4.25 (3–5)	3.80 (3–5)	(1/6)	(2/6)	(0/6)	3.60 (2–5)
Childhood	6	6.83 ± 2.99	41.83 ± 12.04	(2/6)	(5/6)	549 (296–796)	(6/6)	4.17 (3–5)	3.33 (2–4)	(4/6)	(4/6)	(3/6)	3.33 (2–4)
Juvenile	23	15.05 ± 2.50	27.09 ± 12.54	(7/23)	(11/22)	317 (189–642)	(23/23)	4.87 (4–5)	2.30 (1–4)	(6/23)	(6/23)	(3/23)	1.48 (1–3)
Adult	22	31.50 ± 4.50	49.00 ± 9.33	(4/13)	(6/22)	290 (115–628)	(21/21)	4.86 (4–5)	2.57 (1–4)	(7/21)	(7/21)	(8/21)	1.62 (0–4)
Late Onset	6	52.17 ±7.99	61.33 ± 10.23	(0/4)	(5/6)	332 (131–911)	(3/5)	5.00 (5–5)	2.40 (1–4)	(5/5)	(1/5)	(5/5)	2.00 (0–4)
Asymptomatic	2	N/A	30.00 ± 22.63	(0/2)	(2/2)	238 ^a^	(0/2)	5.00 (5–5)	1 (1–1)	(0/2)	(0/2)	(0/2)	0 (0–0)

^a^ CTG size of only one of the two patients available. Age of onset and sampling is given as mean ± SD; MRC scale, MIRS scale, mRS scale and ePAL are given as mean (range); Abbreviations: MRC = Medical Research Council; MIRS = Muscular Impairment Rating Scale; NVM = nocturnal mechanical ventilation; mRS = modified Rankin Scale; ePAL = estimated progenitor CTG size. N/A = not applicable.

**Table 3:** Clinical characteristics of childhood cases.

Patient	Methyl CTCF1/2	Age of Onset (Years)	Age at Sampling (Years)	Gender	ePAL (CTGs)	Myotonia	Facial Weakness	Axial Weakness	Limb Weakness	MIRS	Cardiac Involvement	NVM	CNS Involvement	Hypersomnolence	Cataracts	mRS	DM1-ACTIV
P7	yes/no	6	56	male	756	yes	moderate	moderate	severe distal	4	pacemaker	yes	Moderate learning disability	yes	yes	4	9
P8	no/no	7	43	male	296	yes	mild	mild	mild distal	3	no	yes	no	no	no	2	25
P9	no/no	2	20	male	476	yes	moderate	mild	mild distal	2	1°AV block	no	moderate learning disability	no	no	4	22
P10	yes/no	6	40	female	324	yes	severe	moderate	mild distal	3	no	no	severe cognitive delay	yes	yes	3	14
P11	no/no	10	44	male	644	yes	mild	moderate	mild proximal + distal	4	1°AV block	yes	no	no	yes	3	38
P12	yes/no	10	48	male	796	yes	moderate	moderate	severe proximal + distal	4	pacemaker	yes	severe cognitive delay	yes	no	4	14

Abbreviations: Methyl CTCF1/2 = methylated at the CTCF1 and CTCF2 site; ePAL = estimated progenitor allele size; AV = atrioventricular; MIRS = Muscular Impairment Rating Scale mRS = modified Rankin Scale; NMV = nocturnal mechanical ventilation; CNS = central nervous system.

## Supplementary Materials

The following supporting information can be downloaded at: https://www.mdpi.com/article/10.3390/biomedicines10061372/s1, Figure S1. The location of the primers and CpGs investigated in this study for the five annotated regions. Table S1. Primer combinations and thermocycler settings. Table S2. DNA methylation analysis of CpGi 74 in blood samples across the clinical DM1 subtypes. Table S3. DNA methylation analysis of CpGi 43 in blood across the DM1 clinical subtypes. Table S4. DNA methylation analysis of CpGi 36 in blood across the DM1 clinical subtypes. Table S5. DNA methylation analysis of CTCF1 in blood across the DM1 clinical subtypes. Table S6. DNA methylation analysis of CTCF2 in blood across the DM1 clinical subtypes. Table S7. Clinical characteristics of congenital cases. Table S8. Clinical characteristics of juvenile cases. Table S9. Logistic regression model ePAL/mode vs. Methylation status. Table S10. DNA methylation analysis of CpGi 74 in lymphoblastoids. Table S11. DNA methylation analysis of CpGi 43 in lymphoblastoids. Table S12. DNA methylation analysis of CpGi 36 in lymphoblastoids. Table S13. DNA methylation analysis of CTCF1 in lymphoblastoids. Table S14. DNA methylation analysis of CTCF2 in lymphoblastoids. Table S15. DNA methylation analysis of CpGi 74 in skin and skin-derived cells. Table S16. DNA methylation analysis of CpGi 74 in muscle and muscle-derived cells. Table S17. DNA methylation analysis of CpGi 36 in skin and skin-derived cells. Table S18. DNA methylation analysis of CpGi 36 in muscle and muscle-derived cells. Table S19. DNA methylation analysis of CpGi 43 in skin and skin-derived cells. Table S20. DNA methylation analysis of CpGi 43 in muscle and muscle-derived cells. Table S21. DNA methylation analysis of CTCF1 in skin and skin-derived cells. Table S22. DNA methylation analysis of CTCF1 in muscle and muscle-derived cells. Table S23. CTG expansion sizes in blood and muscle of the patients providing a muscle biopsy. Table S24. DNA methylation analysis of CTCF2 in skin and skin-derived cells. Table S25. DNA methylation analysis of CTCF2 in muscle and muscle-derived cells.
