# Supplementary material for: An Integrative Analysis of DNA Methylation Pattern in Myotonic Dystrophy Type 1 Samples Reveals a Distinct DNA Methylation Profile between Tissues and a Novel Muscle-Associated Epigenetic Dysregulation
Source: Biomedicines. 2022 Jun 10;10(6):1372. doi: 10.3390/biomedicines10061372 (PMC9220235; doi:10.3390/biomedicines10061372)
Supplement: Supplementary file 1 [file biomedicines-10-01372-s001.zip › biomedicines-1716812-supplementary v5 updated.pdf]

Supplemental DATA.

(A)

CpGi 74-F-1 →

GTAAGTAATGGAGCCAGCCTTGGGCAGTGGAGAGAGCGGGGCGCCC

CCGGCCGAGCCTGTGCCCGCAGCCTCGGGCTCCTCCTCCTCCTCTT

CGCCGCTCCGCTCCCCATCAGCACCGGCTGCTGTCGCCGCCTCCTC

CpGi 74-F-2 →

TGCCCTTGTGGTGTAGGGGTCAAAGGCCACAGCGTTGACCCAGGAC

1

TTGTGGCCATGGCCTCGAGCCACCACGCGGCCCTCGGTGAAGGACC

2 3 4 5 6 7

ACACGGTGACCAGGTCATCTTCGCCACCCGTCACCACGTAGCGGCC

GTCAGGGCTCCAGCACACACACAGCAGGCCCCCAAAGTAGCTCTTC

8 9 10 11

ATGAGCCCACGCAGGAGCATGGAGTCGAAGTGGAAGACGCGCAGGC

12 13 14

AGCCATCCTGGCTCACACAGGCCAGGTGCCGGCCATCGGGCGAGAA

15 16 17 18

GGCGAACTCGTTGAGGGGCCCCTCACCCACCGCCCACTTGGCCAGC

19 ← CpGi 74-R-2 ← CpGi 74-R-1

GGGTTGCGGGGTGCCTTGCTCTTGGCAGCATAGACAGAGAAGC

(B)

CpG 43i-F-1 →

GATAGTGAGACAGAGTGGAGACGGCGGGAAAACAAAAGGGCTCGCCCAG

ACCCAACTCCACCCGCTTCTGCACCCAGCCGTGGCCCCGCCCCACCTGC

CpGi 43-F-2 →

CACACCACGCCCATTGGTCCCAAGCCCCGCCTCCAGCCCAGCCCTAGGT

TCTAAGGCTCGGTCATTCATCAATTTCTAAGGCCCCGCCCCAACCCCTA

1 2 3 4 5

TGCCCCGCCCACCACGAGTCAAGTCAGGCTCCCGCCCGGTTCGGCTTAC

6 7 8 9 10 11 12

CTCGCTGAACGCCCCGCGTCCGATCACCTTCAGAATCTCGAAGTCGTCC

13 14

CTCTGCAGTCGGACCTCCTTAAGCCTCACCACGATGGGCTCCGCTGGGG

15 ← CpGi 43-R-2

GGGTGGTGGGGGAAAAGAACCGAGGGTCACCAGAAAGGGCACTGGAGAC

AAGGGGGAAAGCCCCACCCTCTGTCTGTCTCCCCTTCTCTCTGCCTCTC

AGCTTCACCCTAGGACTGTCTGCTTCCCAGGGGCTTCCCCACATAAACA

← CpGi 43-R-1

CCGTGTAAGGTTCTGGGGGCCAAAAATGCC

(C)

CpGi 36-F-1 →

GCAGGTGCTCTGGGGAATGAGTGATTCAGGACCCCAGAAGGTA

GGCACTGTCCTTACTCCAACTTTATGGAGGGAGCATGGGGAGG

CpGi 36-F-2 →

TTCCCGCAGCCGAGCAGGGGCCACAGGTACCTACCCCGCCCCC

1 2

GCTCACCATGGCAGTGAGCCCGTCCTCCACCAAGTCGAAGTTG

3 4 5

CATGTGTCGGTGGCACCTTCGAAATCCGGTGTAAAGGGGGGCA

6 7 8

CGCTGTCCCGGAGACCATCCCAGTCGAGGCCAAAGAAGAAGGG

9 10 11 12

ATGTGTCCGGAAGTCGCCTGCTCCACCCCGGCCCAGCCGTGTC

13 14 15

TCCGGGGGACACAGCAACCGCTGAATGAAGTCTCGAGCCTCCT

16 17 ← CpGi 36-R-2

CAGGGACCCCTTCGTCCACCAGCGGCAGAGAGAGGTGCTCCTG

← CpGi 36-R-1

CTCAGAGGGAGAGGAGGCGATAGCCTGGGAGCG

(D)

CTCF1-F-1 → CTCF1-F-2 →

TGCCGCCGCCCTGGGCTGCATTGGGTTGGTGGCCCACGCCGGC

1 2 3 4

CAACTCACCGCAGTCTGGCGCCGCCCAGGAGCCGCCCGCGCTC

5 6 7

CCTGAACCCTAGAACTGTCTTCGACTCCGGGGCCCCGTTGGAA

8 9 10 11 12

GACTGAGTGCCCGGGGCACGGCACAGAAGCCGCGCCCACCGCC

13 14 15 16

TGCCAGTTCACAACCGCTCCGAGCGTGGGTCTCCGCCCAGCTC

17 18 19 20

CAGTCCTG*TGATCCGGGCCCGCCCCCTAGCGGCCGGGGAGGGA*

21 22 23 24 25 ← CTCF1-R-2

*GGGGCCGGGTCCGCG*GCCGGCGAACGGGGCTCGAAGGGTCCTT

← CTCF1-R-1

GTAGCCGGGAATGCTGCTGCTGCTGCTGCTGCTGCTGCTG

CTGCTGCTGCTGCTGCTGCTGCTGCTGCTGGGGGGATCACAGA

(E)

CTGCTGCTGCTGCTGCTGCTGCTGCTGCTGCTGCTGCTGCTGC

TGCTGCTGCTGCTGGGGGGATCACAGACCATTTCTTTCTTTCG

CTCF2-F-1 → CTCF2-F-2 →

GCCAGGCTGAGGCCCTGACGTGGATGGGCAAACTGCAGGCCTG

1 2 3 4

GGAAGGCAGCAAGCCGGGCCGTCCGTGTTCCATCCTCCACGCA

5 6

CCCCCACCTATCGTTGGTTCGCAAAGTGCAAAGCTTTCTTGTG

7 8 9 10

CATGACGCCCTGCTCTGGGGAGCGTCTGGCGCGATCTCTGCCT

11 ← CTCF2-R

GCTTACTCGGGAAATTTGCTTTTGCCAAA

Figure S1. The location of the primers and CpGs investigated in this study for the five annotated regions: (A) CpGi 74, (B) CpGi 43, (C) CpGi 36, (D) CTCF1 and (E) CTCF2. Red letters indicate the external primers used (PCR1), blue highlight indicates the internal primers (PCR2), green letters are used for the CTG expansion and yellow highlight indicate the CpGs present. The numbers correspond to the CpGs analysed in this study. The other CpGs were not readable.

Table S1. Primer combinations and thermocycler settings

Primer Name	*Sequence (5'-3')*	*Tm*	*Cycles*
Long PCR			
DM-C	AACGGGGCTCGAAGGGTCCT	63.5	28
DM-DR	CAGGCCTGCAGTTTGCCCATC		
Methylation			
CpGi 74-F-1	GGTAAGTAATGGAGTTAGTTT	57	40
CpGi 74-R-1	ACTTCTCTATCTATACTACCA		
CpGi 74-F-2	GTGTAGGGGTTAAAGGTTATAG	55	40
CpGi 74-R-2	CTATCTATACTACCAAAAACAA		
CpGi 43-F-1	GATAGTGAGATAGAGTGGAG	55	40
CpGi 43-R-1	AACATTTTTAACCCCCAAAAC		
CpGi 43-F-2	AGTTTAGTTTTAGGTTTTAAGGT	55	40
CpGi 43-R-2	CTTATCTCCAATACCCTTTCTAA		
CpGi 36-F-1	GGGAATGAGTGATTTAGGATTT	57	40
CpGi 36-R-1	CTCTCCCTCTAAACAAAAACACCT		
CpGi 36-F-2	AGTAGGGGTTATAGGTATTTATTT	57	40
CpGi 36-R-2	TCTAAACAAAAACACCTCTCTCTAC		
CTCF1-F-1	TGTYGTYGTTTTGGGTTGTATTG	57	40
CTCF1-R-1	TTCCYGACTACAAAAACCCTTYG		
CTCF1-F-2	GTTGTATTGGGTTGGTGGTTTA	57	40
CTCF1-R-2	CTACAAAAACCCTTYGAACCC		
CTCF2-F-1	TTYGGTTAGGTTGAGGTTT	57	40
CTCF-2-R	TTAACAAAAACAAATTTCCC		
CTCF2-F-2	TAAATTGTAGGTTTGGGAAG	57	40

1 and 2 in the primer name correspond to PCR 1 and 2 in the nested PCR. Tm= melting temperature

Table S2. DNA methylation analysis of CpGi 74 in blood samples across the clinical DM1 subtypes.

	CpG →	*1*	*2*	*3*	*4*	*5*	*6*	*7*	*8*	*9*	*10*	*11*	*12*	*13*	*14*	*15*	*16*	*17*	*18*	*19*
Congenital	**P1**	1	1	1	1	1	1	1	1	1	1	1	1	1	1	1	1	1	1	1
	**P2**	1	1	1	1	1	1	1	1	1	1	1	1	1	1	1	1	1	1	1
	**P3**	1	1	1	1	1	1	1	1	1	1	1	1	1	1	1	1	1	1	1
	**P5**	1	1	1	1	1	1	1	1	1	1	1	1	1	1	1	1	1	1	1
Childhood	**P7**	1	1	1	1	1	1	1	1	1	1	1	1	1	1	1	1	1	1	1
	**P8**	1	1	1	1	1	1	1	1	1	1	1	1	1	1	1	1	1	1	1
	**P9**	1	1	1	1	1	1	1	1	1	1	1	1	1	1	1	1	1	1	1
	**P10**	1	1	1	1	1	1	1	1	1	1	1	1	1	1	1	1	1	1	1
	**P11**	1	1	1	1	1	1	1	1	1	1	1	1	1	1	1	1	1	1	1
Juvenile	**P13**	1	1	1	1	1	1	1	1	1	1	1	1	1	1	1	1	1	1	1
	**P16**	1	1	1	1	1	1	1	1	1	1	1	1	1	1	1	1	1	1	1
	**P18**	1	1	1	1	1	1	1	1	1	1	1	1	1	1	1	1	1	1	1
	**P19**	1	1	1	1	1	1	1	1	1	1	1	1	1	1	1	1	1	1	1
	**P23**	1	1	1	1	1	1	1	1	1	1	1	1	1	1	1	1	1	1	1
	**P31**	1	1	1	1	1	1	1	1	1	1	1	1	1	1	1	1	1	1	1
Adult	**P36**	1	1	1	1	1	1	1	1	1	1	1	1	1	1	1	1	1	1	1
	**P37**	1	1	1	1	1	1	1	1	1	1	1	1	1	1	1	1	1	1	1

	**P38**	1	1	1	1	1	1	1	1	1	1	1	1	1	1	1	1	1	1	1
	**P40**	1	1	1	1	1	1	1	1	1	1	1	1	1	1	1	1	1	1	1
	**P41**	1	1	1	1	1	1	1	1	1	1	1	1	1	1	1	1	1	1	1
	**P50**	1	1	1	1	1	1	1	1	1	1	1	1	1	1	1	1	1	1	1
	**P51**	1	1	1	1	1	1	1	1	1	1	1	1	1	1	1	1	1	1	1
	**P52**	1	1	1	1	1	1	1	1	1	1	1	1	1	1	1	1	1	1	1
	**P53**	1	1	1	1	1	1	1	1	1	1	1	1	1	1	1	1	1	1	1
	**P54**	1	1	1	1	1	1	1	1	1	1	1	1	1	1	1	1	1	1	1
	**P55**	1	1	1	1	1	1	1	1	1	1	1	1	1	1	1	1	1	1	1
Late-onset	**P56**	1	1	1	1	1	1	1	1	1	1	1	1	1	1	1	1	1	1	1
	**P57**	1	1	1	1	1	1	1	1	1	1	1	1	1	1	1	1	1	1	1
	**P58**	1	1	1	1	1	1	1	1	1	1	1	1	1	1	1	1	1	1	1
	**P59**	1	1	1	1	1	1	1	1	1	1	1	1	1	1	1	1	1	1	1
	**P60**	1	1	1	1	1	1	1	1	1	1	1	1	1	1	1	1	1	1	1
	**P61**	1	1	1	1	1	1	1	1	1	1	1	1	1	1	1	1	1	1	1
Asymptomatic	**P65**	1	1	1	1	1	1	1	1	1	1	1	1	1	1	1	1	1	1	1
Control	**C1**	1	1	1	1	1	1	1	1	1	1	1	1	1	1	1	1	1	1	1
	**C2**	1	1	1	1	1	1	1	1	1	1	1	1	1	1	1	1	1	1	1
	**C3**	1	1	1	1	1	1	1	1	1	1	1	1	1	1	1	1	1	1	1
	**C4**	1	1	1	1	1	1	1	1	1	1	1	1	1	1	1	0.75	1	1	1

Range 0 to 1 indicates 0 to 100% methylation.

Table S3. DNA methylation analysis of CpGi 43 in blood across the DM1 clinical subtypes

	CpG →	***1***	***2***	***3***	***4***	***5***	***6***	***7***	***8***	***9***	***10***	***11***	***12***	***13***	***14***	***15***
Congenital	**P1**	0	0	0	0	0	0	0	0	0	0	0	0	0	0	0.2
	**P2**	0	0	0	0	0	0	0	0	0	0	0	0	0	0	0.2
	**P3**	0	0	0	0	0	0	0	0	0	0	0	0	0	0	0
	**P4**	0	0	0	0	0	0	0	0	0	0	0	0	0	0	0
	**P5**	0	0	0	0	0	0	0	0	0	0	0	0	0	0	0.5
	**P6**	0	0	0	0	0	0	0	0	0	0	0	0	0	0	0.2
Childhood	**P7**	0	0	0	0	0	0	0	0	0	0	0	0	0	0	0
	**P8**	0	0	0	0	0	0	0	0	0	0	0	0	0	0	0
	**P9**	0	0	0	0	0	0	0	0	0	0	0	0	0	0	0.3
	**P10**	0	0	0	0	0	0	0	0	0	0	0	0	0	0	0
	**P11**	0	0	0	0	0	0	0	0	0	0	0	0	0	0	0
	**P12**	0	0	0	0	0	0	0	0	0	0	0		0	0	0
Juvenile	**P13**	0	0	0	0	0	0	0	0	0	0	0	0	0	0	0
	**P16**	0	0	0	0	0	0	0	0	0	0	0	0	0	0	0
	**P18**	0	0	0	0	0	0	0	0	0	0.2	0	0.1	0.2	0.4	0.5
	**P19**	0	0	0	0	0	0	0	0	0	0	0	0	0	0	0.4
	**P20**	0	0	0	0	0	0	0	0	0	0	0	0	0	0	0
	**P23**	0	0	0	0	0	0	0	0	0	0	0	0	0	0	0
	**P31**	0	0	0	0	0	0	0	0	0	0	0	0	0	0	0
Adult	**P36**	0	0	0	0	0	0	0	0	0	0	0	0	0	0	0
	**P37**	0	0	0	0	0	0	0	0	0	0	0	0	0	0	0
	**P38**	0	0	0	0	0	0	0	0	0	0	0	0	0	0	0
	**P40**	0	0	0	0	0	0	0	0	0	0	0	0	0	0	0.1
	**P41**	0	0	0	0	0	0	0	0	0	0	0	0	0	0	0
	**P50**	0	0	0	0	0	0	0	0	0	0	0	0	0	0	0.5
	**P51**	0	0	0	0	0	0	0	0.2	0	0.25	0	0	0	0	0.5
	**P52**	0	0	0	0	0	0	0	0	0	0	0		0.2	0.3	0.45
	**P53**	0	0	0	0	0	0	0	0	0	0	0		0	0	0.15
	**P54**	0	0	0	0	0	0	0	0	0	0	0	0	0	0.2	0.3

	P55	0	0	0	0	0	0	0	0	0	0	0	0	0	0	0
Late-onset	P56	0	0	0	0	0	0	0	0	0	0	0	0	0	0	0
	P57	0	0	0	0	0	0	0	0	0	0	0	0	0	0	0.4
	P58	0	0	0	0	0	0	0	0	0	0	0	0	0	0	0
	P59	0	0	0	0	0	0	0	0	0	0	0	0	0	0	0
	P60	0	0	0	0	0	0	0	0	0	0.2	0	0	0.2	0.3	0.5
	P61	0	0	0	0	0	0	0	0	0	0	0	0	0	0	0
Asymptomatic	P64	0	0	0	0	0	0	0	0	0	0	0.2	0	0.3	0.4	0.6
	P65	0	0	0	0	0	0	0	0	0	0	0	0	0	0	0.1
Control	C1	0	0	0	0	0	0	0	0	0	0	0	0	0	0	0
	C2	0	0	0	0	0	0	0	0	0	0	0	0	0	0.2	0.2
	C3	0	0	0	0	0	0	0	0	0	0	0	0	0	0	0

Range 0 to 1 indicates 0 to 100% methylation. NA = not available

Table S4. DNA methylation analysis of CpGi 36 in blood across the DM1 clinical subtypes

	CpG →	***1***	***2***	***3***	***4***	***5***	***6***	***7***	***8***	***9***	***10***	***11***	***12***	***13***	***14***	***15***	***16***	***17***
Congenital	**P1**	1	1	1	1	1	1	1	1	1	1	0.9	1	1	1	0.9	0.9	0.9
	**P2**	1	1	1	1	1	1	1	1	1		1	1	1	1	1	1	1
	**P3**	1	1	1	1	1	0.5	1	1	1	1	1	1	1	1	0.9	0.9	1
	**P4**	1	1	1	1	1	1	1	1	1	1	1	1	1	1	1	1	1
	**P5**	1	1	1	1	1	1	1	1	1	1	1	1	1	1	1	1	1
Childhood	**P7**	1	1	1	1	1	1	1	1	1	1	1	1	1	1	1	1	1
	**P8**	1	1	1	1	1	1	1	1	1	1	1	1	1	1	1	1	1
	**P9**	1	1	1	1	1	1	1	1	1	1	1	1	1	1	1	1	1
	**P10**	1	1	1	1	1	1	1	1	1	1	1	1	1	1	1	1	1
	**P11**	1	1	1	1	1	1	1	1	1	1	1	1	1	1	1	1	1
	**P12**	1	1	1	1	1	1	1	1	1	1	1	1	1	1	1	1	1
Juvenile	**P13**	1	1	1	1	1	1	1	1	1	1	1	1	1	1	0.8	0.9	1
	**P16**	1	1	1	1	1	1	1	1	1	1	1	1	1	1	1	1	1
	**P19**	1	1	1	1	1	1	1	1	1	1	1	1	1	1	1	1	1
	**P20**	1	1	1	1	1	0.75	1	1	1	1	1	1	1	1	0.9	1	1
	**P23**	1	1	1	1	1	1	1	1	1	1	1	1	1	1	1	1	1
	**P31**	1	1	1	1	1	1	1	1	1	1	1	1	1	1	1	1	1
Adult	**P36**	1	1	1	1	1	1	1	1	1	1	1	1	1	1	1	1	1
	**P37**	1	1	1	1	1	1	1	1	1	1	1	1	1	1	0.9	1	1
	**P38**	1	1	1	1	1	1	1	1	1	1	1	1	1	1	1	1	1
	**P40**	1	1	1	1	1	1	1	1	1	1	1	1	1	1	1	1	1
	**P41**	1	1	1	1	1	1	1	1	1	1	1	1	1	1	0.9	1	1
	**P50**	1	1	1	1	1	1	1	1	1	1	1	1	1	1	1	1	1
	**P51**	1	1	1	1	1	1	1	1	1	1	1	1	1	1	1	1	1
	**P52**	1	1	1	1	1	1	1	1	1	1	1	1	1	1	1	1	1
	**P53**	1	1	1	1	1	1	1	1	1	1	1	1	1	1	1	1	1
	**P54**	1	1	1	1	1	1	1	1	1	1	1	1	1	1	1	1	1
	**P55**	1	1	1	1	1	1	1	1	1	1	1	1	1	1	1	1	1
Late-onset	**P56**	1	1	1	1	1	1	1	1	1	1	1	1	1	1	1	1	1
	**P57**	1	1	1	1	1	0.75	1	1	1	1	1	1	1	1	1	1	1
	**P58**	1	1	1	1	1	1	1	0.8	1	1	1	1	1	1	0.9	0.9	1
	**P59**	1	1	1	1	1	1	1	1	1	1	1	1	1	1	1	1	1
	**P60**	1	1	1	1	1	1	1	1	1	1	1	1	1	1	1	1	1
	**P61**	1	1	1	1	1	1	1	1	1	1	1	1	1	1	1	1	1
Asymptomatic	**P64**	1	1	1	1	1	1	1	0.9	1	1	1	1	1	1	0.9	0.9	1
	**P65**	1	1	1	1	1	1	1	1	1	1	1	1	1	0.9	0.9	0.9	1
Control	**C1**	1	1	1	1	1	1	1	1	1	1	1	1	1	1	0.9	1	1

**C2**	1	1	1	1	1	1	1	1	1	1	1	1	1	1	1	1	1
**C3**	1	1	1	1	1	1	1	1	1	1	1	1	1	1	1	1	1
**C4**	1	1	1	1	1	1	1	1	1	1	1	1	1	1	0.9	0.9	1

Range 0 to 1 indicates 0 to 100% methylation.

Table S5. DNA methylation analysis of CTCF1 in blood across the DM1 clinical subtypes

	CpG →	*1*	*2*	*3*	*4*	*5*	*6*	*7*	*8*	*9*	*10*	*11*	*12*	*13*	*14*	*15*	*16*	*17*	*18*	*19*	*20*	*21*	*22*	*23*	*24*	*25*
Co	**P1**	NA	NA	0.15	0.25	0.25	0.25	0.25	0.5	0.5	0.6	0.65	1	0.65	0.65	0.5	0.75	0.75	NA	NA	NA	NA	NA	NA	NA	NA
	**P1-2**	0.25	0.25	0.25	0.4	0.25	0.25	0.25	0.4	0.25	0.5	0.5	0.4	0.4	0.5	0.4	0.65	0.6	0.6	0.5	0.6	0.9	0.9	0.9	0.9	NA
	**P2**	NA	0.15	0.15	0.25	0.25	0.25	0.25	0.4	0.4	0.5	0.5	0.5	0.5	0.5	0.5	1	0.8	1	0.5	0.75	0.9	0.9	0.75	0.75	NA
	**P3**	0	0.15	0.25	0.15	0.15	0.25	0.25	0.25	0.35	0.4	0.4	0.4	0.4	0.4	0.55	0.6	0.6	0.5	0.5	0.75	0.75	0.75	0.75	0.75	0.75
	**P4**	NA	0.25	0.25	0.25	0.25	0.25	0.25	0.3	0.4	0.5	0.5	0.5	0.4	0.4	0.4	0.75	0.75	0.75	0.5	0.5	NA	NA	NA	NA	NA
	**P5**	1	1	1	1	1	1	1	1	0.45	1	1	1	1	1	1	1	1	1	0.6	0.75	1	0.75	1	1	1
	**P6**	0.25	0.25	0.25	0.4	0.25	0.25	0.25	0.4	0.25	0.25	0.25	0.25	0.4	0.4	0.25	0.5	0.5	0.6	0.5	0.6	0.8	0.9	1	1	NA
Ch	**P7**	1	1	1	0.6	0.75	0.75	0.75	0.75	0.75	0.8	0.75	0.75	0.85	0.85	0.75	0.85	0.85	0.85	0.9	0.9	0.9	0.9	0.9	0.9	0.9
	**P8**	0	0	0	0	0	0	0	0	0	0	0	0.25	0	0	0	0	0	0	0	0	NA	NA	NA	NA	NA
	**P9**	0	0	0	0	0	0	0	0	0	0	0	0.25	0	0	0	0	0	0	0	0	NA	NA	NA	NA	NA
	**P10**	NA	0.25	0.5	0.5	0.25	0.25	0.25	0.25	0.25	0.4	0.5	0.45	0.45	0.5	0.4	0.75	0.75	0.75	0.5	0.75	0.75	0.75	0.75	0.75	0.75
	**P11**	0	0	0	0	0	0	0	0	0	0	0	0	0	0	0	0	0	0	0	0	0	0	0	0	0
	**P12**	NA	0.5	0.75	0.5	0.5	0.5	0.5	0.5	0.5	0.6	0.75	0.55	0.5	0.5	0.5	0.75	0.5	0.55	0.4	0.5	0.75	0.8	0.75	0.8	0.8
Juv	**P13**	0	0	0	0	0	0	0	0	0	0	0	0	0	0	0	0	0	0	0	0	0	0	0	0	0
	**P14**	NA	0	0	0	0	0.25	0	0	0.25	0	0	0	0	0	0	0	0	0	0	0	0	0	0	0	0
	**P15**	NA	0	0	0	0	0	0	0	0	0	0	0	0	0	0	0	NA	NA	NA	NA	NA	NA	NA	NA	NA
	**P16**	0	0	0	0	0	0	0	0	0	0	0	0.5	0	0	0	0	0	0	0	0	NA	NA	NA	NA	NA
	**P17**	0	0	0	0	0	0	0	0	0	0	0	0.25	0	0	0	0	0	0	0	0	NA	NA	NA	NA	NA
	**P18**	0	0	0	0	0	0	0	0	0	0	0	0	0	0	0	0	0	0	0	0	0	0	0	0	0
	**P19**	NA	0.25	0.25	0.25	0.25	0.25	0.25	0.4	0.5	0.5	0.5	0.5	0.5	0.5	0.5	0.8	0.9	0.9	0.5	0.6	0.9	1	0.75	0.75	NA
	**P20**	NA	0.15	0.15	0.25	0.25	0.25	0.25	0.4	0.4	0.5	0.5	0.5	0.5	0.5	0.5	0.75	0.75	0.75	0.75	0.5	0.4	0.9	0.9	0.5	0.9
	**P21**	0	0	0	0	0	0	0	0	0	0	0	0	0	0	0	0	0	0	0	0	0	0	0	0	0
	**P22**	NA	0	0	0	0	0	0	0	0	0	0	0	0	0	0	0	0	0	0	0	0	0	0	0	0
	**P23**	NA	0	0	0	0	0	0	0	0	0	0	0	0	0	0	0	0	0	0	0	0	0	0	0	0
	**P24**	NA	0.05	0.05	0.05	0.05	0	0	0	0	0	0	0	0	0	0	0	0	0	0	0	0	NA	NA	NA	NA
	**P25**	NA	0	0	0	0	0	0	0	0	0	0	0	0	0	0	0	0	0	0	0	0	NA	NA	NA	NA
	**P26**	NA	0	0	0	0	0	0	0	0	0	0	0	0	0	0	0	0	0	0	0	0	NA	NA	NA	NA
	**P27**	NA	0	0	0	0	0	0	0	0	0	0	0	0	0	0	0	0	0	0	0	0	NA	NA	NA	NA
	**P28**	0	0	0	0	0	0	0	0	0	0	0	0	0	0	0	0	0	0	0	0	0	0	0	NA	NA
	**P29**	0.05	0.05	0.05	0.05	0	0	0	0	0	0	0	0	0	0	0	0	0	0	0	0	0	0	0	NA	NA
	**P30**	0.05	0.05	0.05	0	0	0	0	0	0	0	0	0	0	0	0	0	0	0	0	0	0	0	0	NA	NA
	**P31**	0.4	0.4	0.25	0.45	0.6	0.75	0.75	0.75	0.7	0.8	0.75	0.8	0.8	0.8	0.75	0.75	0.85	0.85	0.85	1	1	0.9	0.75	1	1
	**P32**	0	0	0	0	0	0	0	0	0	0	0	0	0	0	0	0	0	0	0	0	0	0	NA	NA	NA
	**P33**	0	0	0	0	0	0	0	0	0	0	0	0	0	0	0	0	0	0	0	0	0	0	NA	NA	NA
	**P34**	0	0	0	0	0	0	0	0	0	0	0	0	0	0	0	0	0	0	0	NA	NA	NA	NA	NA	NA
	**P35**	0	0	0	0	0	0	0	0	0	0	0	0	0	0	0	0	0	0	0	NA	NA	NA	NA	NA	NA
A	**P36**	0	0	0	0	0	0	0	0	0	0	0	0	0	0	0	0	0	0	0	0	0	0	0	0	NA
	**P37**	0	0	0	0	0	0	0	0	0	0	0	0	0	0	0	0	0	0	0	0	NA	NA	NA	NA	NA
	**P38**	0	0	0	0	0	0	0	0	0	0	0	0.25	0	0	0	0	0	0	0	0	NA	NA	NA	NA	NA
	**P39**	0	0	0	0	0	0	0	0	0	0	0	0.25	0	0	0	0	0	0	0	0	NA	NA	NA	NA	NA
	**P40**	0	0	0	0	0	0	0	0	0	0	0	0.25	0	0	0	0	0	0	0	0	NA	NA	NA	NA	NA
	**P41**	0	0	0	0	0	0	0	0	0	0	0	0	0	0	0	0	0	0	0	0	0	NA	NA	NA	NA
	**P42**	0	0	0	0	0	0	0	0	0	0	0	0	0	0	0	0	0	0	0	0	0	0	0	NA	NA
	**P43**	0	0	0	0	0	0	0	0	0	0	0	0	0	0	0	0	0	0	0	0	0	0	0	NA	NA
	**P45**	0	0	0	0	0	0	0	0	0	0	0	0	0	0	0	0	0	0	0	0	0	0	0	NA	NA
	**P46**	0	0	0	0	0	0	0.25	0	0	0.25	0	0	0	0	0	0	0	0	0	0	0	0	0	0	0
	**P47**	NA	0	0	0	0	0	0	0.1	0	0	0	0	0	0	0	0	0	0	0	0	NA	NA	NA	NA	NA
	**P48**	NA	0	0	0	0	0	0	0	0	0	0	0	0	0	0	0	0	0	0	0	0	NA	NA	NA	NA
	**P49**	NA	0	0	0	0	0	0	0	0	0	0	0	0	0	0	0	0	0	0	0	NA	NA	NA	NA	NA
	**P50**	0.1	0.1	0.1	0.2	0.2	0.2	0.2	0.2	0.2	0.25	0.25	0.25	0.25	0.3	0.25	0.4	0.5	0.5	0.5	0.5	0.6	NA	0.75	0.6	0.5
	**P51**	0	0	0	0	0	0	0	0	0	0	0	0	0	0	0	0	0	0	NA	NA	NA	NA	NA	NA	NA
	**P52**	NA	0	0	0	0	0	0	0	0	0	0	0	0	0	0	0	0	0	0	0	0	0	0	0	0
	**P53**	NA	0	0	0	0	0	0	0	0	0	0	0	0	0	0	0	0	0	0	0	0	0	0	0	0

	**P54**	0	0	0	0	0	0	0	0	0	0	0	0	0	0	0	0	0	0	0	0	0	0	0	0	0
	**P55**	NA	0	0	0	0	0	0	0	0	0	0	0	0	0	0	0	0	0	0	0	0				
LO	**P56**	0	0	0	0	0	0	0	0	0	0	0	0	0	0	0	0	0	0	0	0	0	0	0	0	0
	**P57**	0	0	0	0	0	0	0	0	0	0	0	0.25	0	0	0	0	0	0	0	0	NA	NA	NA	NA	NA
	**P58**	0	0	0	0	0	0	0	0	0	0	0	0.25	0	0	0	0	0	0	0	0	NA	NA	NA	NA	NA
	**P59**	0	0	0	0	0	0	0	0	0	0	0	0.25	0	0	0	0	0	0	0	0	NA	NA	NA	NA	NA
	**P60**	0	0	0	0	0	0	0	0	0	0	0	0.25	0	0	0	0	0	0	0	0	NA	NA	NA	NA	NA
	**P61**	NA	0	0	0	0	0	0.25	0	0	0	0	0	0	0	0	0	0	0	0	0	NA	NA	NA	NA	NA
	**P62**	NA	0	0	0	0	0	0.25	0	0	0	0	0	0	0	0	0	0	0	0	0	NA	NA	NA	NA	NA
	**P63**	NA	0	0	0	0	0	0	0	0	0	0	0	0	0	0	0	0	0	0	0	0				
As	**P64**	0	0	0	0	0	0	0	0	0	0	0	0	0	0	0	0	0	0	0	0	0	0	0	0	0
	**P65**	0	0	0	0	0	0	0	0	0	0	0	0	0	0	0	0	0	0	0	0	0	0	0	0	0
C	**Ctrl 1**	NA	0	0	0	0	0	0	0	0	0	0	0	0	0	0	0	0	0	0	0	0	0	0	0	0
	**Ctrl 2**	0	0	0	0	0	0	0.2	0	0	0	0	0	0	0	0	0	0	0	0	0	0	0	0	0	0
	**Ctrl 3**	NA	0	0	0	0	0	0	0	0	0	0	0.25	0	0	0	0	0	0.25	0	0	0	0	0		0
	**Ctrl 4**	0	0	0	0	0	0	0.2	0	0	0	0	0	0	0	0	0	0	0	0	0	0	0	0	0	0
	**Ctrl 5**	NA	0	0	0	0	0.4	0	0	0	0	0	0	0	0	0.25	0	0	0	0	0	0	0	0	NA	NA
	**Ctrl 6**	NA	0	0	0	0	0.1	0	0	0	0	0	0	0	0	0	0	0	0	0	0	0	0	0	0	0

Range 0 to 1 indicates 0 to 100% methylation. NA = not available. P1-2 = five year follow-up of P1. Co= congenital, Ch= childhood, Juv= juvenile, A= adult, LO= late-onset, As= asymptomatic, C= control

Table S6. DNA methylation analysis of CTCF2 in blood across the DM1 clinical subtypes

	CpG →	***1***	***2***	***3***	***4***	***5***	***6***	***7***	***8***	***9***	***10***	***11***
Congenital	**P1**	0	0	0	0	0	0	0.1	0	0	0	0
	**P1-2**	0	0	0	0	0	0.05	0	0.05	0.1	0.1	0
	**P2**	0	0	0	0.1	0.1	0.15	0.1	0.1	0.15	0.2	0.25
	**P3**	NA	NA	NA	0.05	0.05	0.1	0.05	0	0	0	0.1
	**P4**	0	0	0	0	0	0	0	0.05	0.05	0.05	0.05
	**P5**	NA	NA	NA	0.4	0.25	0.4	0.3	0.4	0.6	0.75	0.75
	**P6**	0	0	0	0.05	0.05	0.1	0.1	0.15	0.25	0.45	0.45
Childhood	**P7**	0	0	0	0	0	0	0	0	0	0	0
	**P8**	0	0	0	0	0	0	0	0	0	0	0
	**P9**	0	0	0	0	0	0	0	0	0	0	0
	**P10**	0	0	0	0	0	0	0	0.05	0.05	0.05	0
	**P11**	0	0	0	0	0	0	0	0	0	0	0
	**P12**	0	0	0	0	0	0	0	0	0	0	0
Juvenile	**P13**	0	0	0	0	0	0	0	0	NA	NA	NA
	**P14**	0	0	0	0.05	0	0.05	0.05	0	0.05	0.05	0
	**P15**	0	0	0	0	0	0	0	0	0	0	0
	**P16**	0	0	0	0	0	0	0	0	0	0	0
	**P17**	0	0	0	0	0	0	0.1	0	0	0	0
	**P18**	0	0	0	0	0	0	0	0	NA	NA	NA
	**P19**	0.05	0.05	0	0.15	0.1	0.15	0.1	0.1	0.2	0.25	0.25
	**P20**	0.05	0.05	0	0.2	0.15	0.2	0.1	0.25	0.45	0.5	0.5
	**P21**	0	0	0	0	0	0	0.5	0	0	0	0
	**P22**	NA	NA	NA	0	0	0	0.2	0	0	0	0
	**P23**	0	0	0	0	0	0.05	0.05	0.05	0.1	0.1	0.1
	**P24**	0	0	0	0	0	0	0	0	0	0	0
	**P25**	0	0	0	0	0	0	0	0	0.05	0.05	0.05
	**P26**	0	0	0	0	0	0	0	0	0	0	0

	**P27**	0	0	0	0.05	0.05	0	0	0	0	0	0
	**P28**	NA	NA	NA	0	0	0	0	0	0	0	0
	**P29**	NA	NA	NA	0	0	0	0.2	0	0	0	0
	**P30**	0	0	0	0.05	0	0	0	0	0	0	0
	**P31**	NA	NA	NA	0.5	0.6	1	0.75	0.75	1	1	1
	**P32**	0	0	0	0	0	0	0	0	0	0	0
	**P33**	0	0	0	0	0	0	0	0	0	0	0
	**P35**	0	0	0	0	0	0	0	0	0	0	0
	**P37**	0	0	0	0	0	0	0	0	0	0	0
	**P38**	0	0	0	0	0	0	0	0	0	0	0
	**P39**	0	0	0	0	0	0	0	0	0	0	0.05
	**P40**	0	0	0	0	0	0	0	0	0	0	0
	**P41**	0	0	0	0	0	0	0	0	0	0	0
	**P42**	0	0	0	0	0	0	0	0	0	0	0
	**P43**	0	0	0	0	0	0	0	0	0	0	0
	**P44**	NA	NA	NA	0	0	0	0.5	0	0	0	0
	**P45**	0	0	0	0	0	0	0	0	0	0	0
	**P48**	0	0	0	0	0	0	0	0	0	0	0
	**P49**	0	0	0	0	0	0	0	0	0	0	0
	**P50**	NA	NA	NA	0	0	0	0	0	0	0	0
	**P51**	NA	NA	NA	0	0	0	0	0	0	0	0
	**P52**	0	0	0	0	0	0	0	0	0	0	0
	**P53**	0	0	0	0	0	0	0	0	0	0	0
	**P54**	0	0	0	0	0	0	0.1	0	0	0	0
	**P55**	0	0	0	0	0	0	0	0	NA	NA	NA
Late-onset	**P56**	0	0	0	0	0	0	0	0	NA	NA	NA
	**P57**	0	0	0	0	0	0	0	0	0	0	0
	**P58**	0	0	0	0	0	0	0	0	0	0	0
	**P59**	0	0	0	0	0	0	0	0	0	0	0
	**P60**	0	0	0	0	0	0	0.05	0	0	0	0
	**P63**	0	0	0	0	0	0	0.1	0	0	0	0
Asymptomatic	**P64**	0	0	0	0	0	0	0	0	NA	NA	NA
	**P65**	0	0	0	0	0	0	0	0	NA	NA	NA
Control	**Ctrl 1**	0	0	0	0	0	0	0	0	0	0	0
	**Ctrl 2**	0	0	0	0	0	0	0	0	0	0	0
	**Ctrl 3**	0	0	0	0	0	0	0	0	0	0	0
	**Ctrl 4**	0	0	0	0	0	0	0	0	0	0	0
	**Ctrl 5**	0	0	0	0	0	0	0	0	0	0	0
	**Ctrl 6**	0	0	0	0	0	0	0	0	0	0	0
	**Ctrl 7**	NA	0	0	0	0	0	0	0	0	0	0

Range 0 to 1 indicates 0 to 100% methylation. NA = not available. P1-2= five year follow-up P1

Table S7. Clinical characteristics of congenital cases

Patient	*Methyl CTCF1/2*	*Age of onset (years)*	*Age at sampling (years)*	*Gender*	*ePAL (CTGs)*	*Myotonia*	*Facial weakness*	*Axial weakness*	*Limb weakness*	*MIRS*	*Cardiac involvement*	*NVM*	*Hyper-somnolence*	*Cataracts*	*mRS*	*DM1-ACTIV*
P1	yes/no	birth	10	female	831	no	mild	moderate	moderate proximal	4	no	no	no	no	3	32
P2	yes/yes	birth	18	male	587	no	severe	mild	severe proximal	5	LAFB	no	U	no	5	1
P3	yes/no	birth	2	male	1011	no	moderate	moderate	NA	4	no	no	no	no	NA	NA
P4	yes/no	birth	16	male	730	yes	moderate	mild	moderate distal	3	no	yes	no	no	3	38

P5	yes/yes	birth	16	male	222	yes	unknown	none	moderate distal	3	no	no	no	no	2	40
P6	yes/yes	birth	15	female	280	no	mild	moderate	moderate proximal	4	no	no	yes	no	3	30

Methyl CTCF1/2= hypermethylated at the CTCF1 and CTCF2 site; ePAL= estimated progenitor allele size; NA = not applicable; U= unknown; AV = atrioventricular; LAFB = left anterior fascicular block; mRS = modified Rankin Scale; MIRS = Muscular Impairment Rating Scale; NMV = nocturnal mechanical ventilation.

Table S8. Clinical characteristics of juvenile cases

Patient	***Methyl CTCF1/2***	***Age of onset (years)***	***Age at sampling (years)***	***Gender***	***ePAL (CTGs)***	***Myotonia***	***Facial weakness***	***Axial weakness***	***Limb weakness***	***MIRS***	***Cardiac involvement***	***NVM***	***Hyper-somnolence***	***Cataracts***	***mRS***	***DM1-ACTIV***
P13	no/no	15	39	female	286	yes	mild	none	mild/moderate proximal+distal	4	LAFB	no	yes	no	2	U
P14	no/no	12	22	male	NA	yes	none	none	none	2	NA	no	no	no	1	40
P15	no/no	18	54	female	392	yes	moderate	U	mild/moderate proximal+distal	4	1º AV block	no	no	yes	3	13
P16	no/no	11	12	male	517	yes	U	U	none	2	no	no	U	no	1	36
P17	no/no	16	19	female	273	yes	mild	U	mild distal	1	no	no	no	no	1	40
P18	no/no	11	16	female	222	yes	none	none	mild distal	2	no	no	no	no	1	40
P19	yes/yes	15	26	female	U	yes	none	none	none	2	no	no	yes	no	1	39
P20	yes/yes	15	24	male	189	yes	none	none	none	2	no	no	no	no	3	37
P21	no/no	12	40	female	428	yes	mild	moderate	mild distal	3	no	yes	yes	no	2	18
P22	no/no	19	37	female	251	yes	mild	none	mild distal	3	no	no	no	no	1	29
P23	no/no	12	14	female	642	yes	mild	none	none	2	no	no	yes	no	1	40
P24	no/no	14	16	female	229	yes	mild	none	none	2	no	no	no	no	1	40
P25	no/no		15	male	235	yes	none	none	none	1	1ºAV block	no	no	no	0	40
P26	no/no	15	16	male	323	yes	mild	none	none	2	no	no	no	no	1	40
P27	no/no	16	37	female	330	yes	mild	mild	mild/moderate proximal+distal	4	1ºAV block	no	yes	yes	2	29
P28	no/no	17	45	male	236	yes	mild	U	mild/moderate proximal+distal	3	1ºAV block + LAFB	yes	no	no	3	24
P29	no/no	18	26	female	254	yes	none	none	none	1	no	no	no	no	1	40
P30	no/no	18	24	male	274	yes	mild	none	mild distal	2	no	no	yes	no	1	33
P31	yes/yes	16	53	female	420	yes	mild	none	mild/moderate proximal+distal	3	no	no	no	yes	2	27
P32	no/no	15	29	male	237	yes	moderate	none	none	2	pacemaker	yes	no	no	3	19
P33	no/no	19	24	male	228	yes	mild	none	mild distal	2	no	no	yes	no	1	37
P34	no/no	13	20	female	368	yes	mild	none	mild distal	2	no	no	yes	no	1	39
P35	no/no	14	15	male	318	yes	mild	none	none	2	no	no	yes	no	1	39

Methyl CTCF1/2= hypermethylated at the CTCF1 and CTCF2 site; ePAL= estimated progenitor allele size; U= unknown; AV = atrioventricular; LAFB = left anterior fascicular block; mRS = modified Rankin Scale; MIRS = Muscular Impairment Rating Scale; NMV = nocturnal mechanical ventilation

Table S9. Logistic regression model ePAL/modal vs. Methylation status

Model	***Variables***	***OR***	***CI***	***p-value***	***N***
1	ePAL versus MetS	1.005	1.002-1.008	0.004	53
2	Modal versus MetS	1.004	1.002-1.007	0.001	46

Significance is set at p= ≤0.05. ePAL= estimated progenitor allele size; MetS= methylation status

Table S10. DNA methylation analysis of CpGi 74 in lymphoblastoids

	CpG →	1	2	3	4	5	6	7	8	9	10	11	12	13	14	15	16	17	18	19
Congenital	**P1**	1	1	1	1	1	1	1	1	1	1	1	1	1	1	1	1	1	1	1
	**P2**	1	1	1	1	1	1	1	1	1	1	1	1	1	1	1	1	1	1	1
	**P4**	1	1	1	1	1	1	1	1	1	1	1	1	1	1	1	1	1	1	1
Childhood	**P7**	1	1	1	1	1	1	1	1	1	1	1	1	1	1	1	1	1	1	1
	**P8**	1	1	1	1	1	1	1	1	1	1	1	1	1	1	1	1	1	1	1
	**P9**	1	1	1	1	1	1	1	1	1	1	1	1	1	1	1	1	1	1	1
	**P10**	1	1	1	1	1	1	1	1	1	1	1	1	1	1	1	1	1	1	1
	**P11**	1	1	1	1	1	1	1	1	1	1	1	1	1	1	1	1	1	1	1
Juvenile	**P13**	1	1	1	1	1	1	1	1	1	1	1	1	1	1	1	1	1	1	1
	**P20**	1	1	1	1	1	1	1	1	1	1	1	1	1	1	1	1	1	1	1
Adult	**P36**	1	1	1	1	1	1	1	1	1	1	1	1	1	1	1	1	1	1	1
	**P38**	1	1	1	1	1	1	1	1	1	1	1	1	1	1	1	1	1	1	1
Late-onset	**P56**	1	1	1	1	1	1	1	1	1	1	1	1	1	1	1	1	1	1	1
Control	**C1**	1	1	1	1	1	1	1	1	1	1	1	1	1	1	1	1	1	1	1
	**C4**	1	1	1	1	1	1	1	1	1	1	1	1	1	1	1	1	1	1	1
	**C5**	1	1	1	1	1	1	1	1	1	1	1	1	1	1	1	1	1	1	1
	**C6**	1	1	1	1	1	1	1	1	1	1	1	1	1	1	1	1	1	1	1

Range 0 to 1 indicates 0 to 100% methylation.

Table S11. DNA methylation analysis of CpGi 43 in lymphoblastoids

	CpG →	1	2	3	4	5	6	7	8	9	10	11	12	13	14	15
Congenital	**P1**	0	0	0	0	0	0	0	0	0	0	0	0	0	0.35	0.3
	**P2**	0	0	0	0	0	0	0	0	0	0	0	0	0	0	0.25
	**P4**	0	0	0	0	0	0	0	0	0	0	0	0	0	0	0.15
Childhood	**P7**	0	0	0	0	0	0	0	0	0	0	0	0	0.1	0	0.2
	**P8**	0	0	0	0	0	0	0	0	0	0	0	0	0	0	0
	**P9**	0	0	0	0	0	0	0	0	0	0	0	0	0	0	0.3
	**P10**	0	0	0	0	0	0	0	0	0	0	0	0	0	0	0
	**P11**	0	0	0	0	0	0	0	0	0	0	0	0	0	0.3	0.6
Juvenile	**P13**	0	0	0	0	0	0	0	0	0	0	0	0	0	0.2	0.2
	**P20**	0	0	0	0	0	0	0	0	0	0	0	0	0	0	0
Adult	**P36**	0	0	0	0	0	0	0	0	0	0	0	0	0	0	0
	**P38**	0	0	0	0	0	0	0	0	0	0	0	0	0	0	0
Late-onset	**P56**	0	0	0	0	0.2	0	0	0	0	0	0	0	0	0.4	0.3
Control	**C1**	0	0	0	0	0	0	0	0	0	0	0	0	0	0	0
	**C4**	0	0	0	0	0	0	0	0	0	0	0	0	0	0.45	0.45
	**C5**	0	0	0	0	0	0	0	0	0	0	0	0	0	0	0
	**C6**	0	0	0	0	0	0	0	0	0	0	0	0	0	0	0

Range 0 to 1 indicates 0 to 100% methylation.

Table S12. DNA methylation analysis of CpGi 36 in lymphoblastoids

	CpG →	*1*	*2*	*3*	*4*	*5*	*6*	*7*	*8*	*9*	*10*	*11*	*12*	*13*	*14*	*15*	*16*	*17*
Congenital	**P2**	1	1	1	1	1	1	1	1	1	1	1	1	1	1	0.8	1	1
	**P4**	1	1	1	1	1	1	1	1	1	1	1	1	1	1	0.9	0.9	1
Childhood	**P7**	1	1	1	1	1	1	1	1	1	1	1	1	1	1	1	1	1
	**P8**	1	1	1	1	1	1	1	1	1	1	1	1	1	1	1	1	1
	**P9**	1	1	1	1	1	1	1	1	1	1	1	1	1	1	1	1	1
	**P10**	1	1	1	1	1	1	1	1	1	1	1	1	1	1	0.9	0.9	1
Juvenile	**P13**	1	1	1	1	1	1	1	1	1	1	1	1	1	1	0.9	0.9	1
Adult	**P36**	1	1	1	1	1	1	1	1	1	1	1	1	1	1	1	0.9	1
	**P38**	1	1	1	1	1	1	1	1	1	1	1	1	1	1	0.9	0.9	1
Late-onset	**P56**	1	1	1	1	1	1	1	1	1	1	1	1	1	1	1	1	1
Control	**C4**	1	1	1	1	1	1	1	1	1	1	1	1	1	1	0.9	0.8	1
	**C5**	1	1	1	1	1	1	1	1	1	0.8	1	1	1	1	0.9	0.9	1
	**C6**	1	1	1	1	1	1	1	0.8	1	1	1	1	1	1	0.8	0.8	1

Range 0 to 1 indicates 0 to 100% methylation.

Table S13. DNA methylation analysis of CTCF1 in lymphoblastoids

	CpG →	*1*	*2*	*3*	*4*	*5*	*6*	*7*	*8*	*9*	*10*	*11*	*12*	*13*	*14*	*15*	*16*	*17*	*18*	*19*	*20*	*21*	*22*	*23*	*24*	*25*
Congenital	**P1**	0.25	0.25	0.25	0.4	0.5	0.5	0.5	0.5	0.5	0.5	0.5	0.5	0.5	0.5	0.5	0.45	0.5	0.5	0.6	0.4	0.6	0.75	0.75	0.75	0.75
	**P2**	0.2	0.2	0.2	0.3	0.3	0.45	0.4	0.4	0.45	0.45	0.5	0.4	0.4	0.5	0.4	0.6	0.55	0.55	0.45	0.6	0.75	0.75	0.75	0.75	0.8
	**P4**	0.1	0.1	0.1	0.1	0.1	0.1	0.2	0.1	0.1	0.1	0.1	0.1	0.1	0.15	0.1	0.1	0.1	0.1	0.1	0.2	0.25	0.25	0.2	0.3	0.25
Childhood	**P7**	0	0	0	0	0	0	0	0	0	0	0	0	0	0	0	0	0	0	0	0	0	0	0	0	0
	**P8**	0	0	0	0	0	0	0	0	0	0	0	0	0	0	0	0	0	0	0	0	0	0	0	0	0
	**P9**	0	0	0	0	0	0	0	0	0	0	0	0	0	0	0	0	0	0	0	0	0	0	0	0	0
	**P10**	0.25	0.25	0.25	0.5	0.4	0.4	0.45	0.5	0.5	0.5	0.5	0.5	0.5	0.65	0.5	0.75	0.5	0.55	0.45	0.55	0.75	0.8	0.8	0.8	0.75
	**P11**	0	0	0	0	0	0	0	0	0	0	0	0	0	0	0	0	0	0	0	0	0	0	0	0	0
Juvenile	**P13**	0	0	0	0	0	0	0	0	0	0	0	0	0	0	0	0	0	0	0	0	0	0	0	0	0
	**P20**	0.2	0.2	0.2	0.4	0.3	0.3	0.3	0.4	0.4	0.35	0.4	0.4	0.4	0.4	0.45	0.5	0.5	0.55	0.5	0.75	0.75	0.75	0.75	0.7	0.7
Adult	**P36**	0	0	0	0	0	0	0	0	0	0	0	0	0	0	0	0	0	0	0	0	0	0	0	0	0
	**P38**	0	0	0	0	0	0	0	0	0	0	0	0	0	0	0	0	0	0	0	0	0	0	0	0	0
Late-onset	**P56**	0	0	0	0	0	0	0	0	0	0	0	0	0	0	0	0	0	0	0	0	0	0	0	0	0
Control	**C1**	0	0	0	0	0	0	0	0	0	0	0	0	0	0	0	0	0	0	0	0	0	0	0	0	0
	**C4**	0	0	0	0	0	0	0	0	0	0	0	0	0	0	0	0	0	0	0	0	0	0	0	0	0
	**C5**	0	0	0	0	0	0	0	0	0	0	0	0	0	0	0	0	0	0	0	0	0	0	0	0	0
	**C6**	0	0	0	0	0	0	0	0	0	0	0	0	0	0	0	0	0	0	0	0	0	0	0	0	0

Range 0 to 1 indicates 0 to 100% methylation.

Table S14. DNA methylation analysis of CTCF2 in lymphoblastoids

	CpG →	*1*	*2*	*3*	*4*	*5*	*6*	*7*	*8*	*9*	*10*	*11*
Congenital	**P2**	NA	0	0	0.1	0.1	0.1	0.1	0.15	0.1	0.15	0.2
	**P4**	NA	0	0	0.1	0.1	0.1	0.1	0.1	0.1	0.15	0.15
	**P7**	NA	0	0	0	0	0.15	0	0	0.05	0.05	0.05
Childhood	**P8**	0	0	0	0	0	0	0	0	0	0	0
	**P9**	NA	0	0	0	0	0	0	0.05	0.05	0.05	0.05
	**P10**	NA	0	0	0.05	0.1	0.1	0.1	0.1	0.1	0.15	0.1
	**P11**	NA	0	0	0	0	0.05	0	0	0	0.5	0.1

	**P13**	0	0	0	0	0	0	0	0	0	0	0
Juvenile	**P20**	NA	0	0	0.1	0.1	0.2	0.05	0.1	0.1	0.1	0.15
	**P36**	0	0	0	0	0	0	0	0	0	0	0
Adult	**P38**	0	0	0	0	0	0	0	0	0	0	0
	**P56**	0	0	0	0	0	0	0	0	0	0	0
Late-onset	**C1**	0	0	0	0	0	0	0	0	0	0	0
Control	**C4**	0	0	0	0	0	0	0	0	0	0	0
	**C5**	0	0	0	0	0	0	0	0	0	0	0
	**C6**	0	0	0	0	0	0	0	0	0	0	0

Range 0 to 1 indicates 0 to 100% methylation. NA = not available

Table S15. DNA methylation analysis of CpGi 74 in skin and skin-derived cells

	CpG →	***1***	***2***	***3***	***4***	***5***	***6***	***7***	***8***	***9***	***10***	***11***	***12***	***13***	***14***	***15***	***16***	***17***	***18***	***19***
Skin																				
Adult	**P37**	1	0.85	1	1	0.7	1	0.75	1	1	1	1	1	1	1	1	0.7	1	1	1
	**P68**	1	0.75	1	1	1	1	1	1	1	1	1	1	1	1	1	0.5	1	1	1
	**P69**	0.75	1	1	1	1	1	1	0.95	1	1	1	1	1	1	1	0.7	1	1	1
Control	**C2**	1	0.75	1	1	1	0.9	0.75	0.9	1	1	1	1	1	1	1	0.6	1	0.8	0.8
	**C10**	1	0.8	1	1	1	0.75	0.8	0.8	1	1	1	1	1	1	1	0.75	1	0.95	0.95
Skin fibroblasts																				
Juvenile	**P13**	1	1	1	1	1	1	1	1	1	1	1	1	1	1	1	0.5	1	1	1
Adult	**P36**	1	1	1	1	1	1	1	1	1	1	1	1	1	1	1	1	1	1	1
	**P38**	1	1	1	1	1	1	1	1	1	1	1	1	1	1	1	1	1	1	1
Control	**C2**	1	1	1	1	1	1	1	1	1	1	1	1	1	1	1	1	1	1	1
	**C6**	1	1	1	1	1	1	1	1	1	1	1	1	1	1	1	1	0.75	1	1
	**C9**	1	1	1	1	1	1	1	1	0.75	1	1	1	0.75	1	1	0.75	1	1	1

Range 0 to 1 indicates 0 to 100% methylation.

Table S16. DNA methylation analysis of CpGi 74 in muscle and muscle-derived cells

	CpG →	***1***	***2***	***3***	***4***	***5***	***6***	***7***	***8***	***9***	***10***	***11***	***12***	***13***	***14***	***15***	***16***	***17***	***18***	***19***
Muscle																				
Juvenile	**P13**	1	0.75	1	1	0.75	1	0.65	1	0.85	0.9	0.9	1	1	1	1	0.5	1	1	1
Adult	**P37**	1	0.75	1	1	0.73	0.9	0.55	0.9	1	1	1	1	1	1	1	0.85	1	1	1
	**P68**	0	0.5	1	0.6	0.75	0.75	0.4	0.83	0.83	0.83	1	0.9	1	1	1	0.6	1	1	1
Late-onset	**P56**	0.68	0.75	1	0.95	0.73	0.9	0.55	0.85	1	0.9	1	1	1	1	1	0.75	1	1	1
	**P67**	0.4	0.75	0.75	1	0.95	1	0.75	1	1	1	1	1	1	1	0.9	0.2	1	1	1
Control	**C6**	1	1	1	1	1	1	1	0.85	1	1	0.5	1	1	1	1	0.5	1	0.7	1
	**C9**	1	0.67	1	1	0.8	1	0.6	0.9	1	1	1	1	1	1	1	0.8	1	0.9	0.9
Myoblasts																				
Adult	**P36**	1	1	1	NA	NA	NA	NA	1	1	NA	NA	1	1	1	1	1	1	1	1
	**P37**	1	1	1	1	1	1	0.75	0.75	1	0.9	0.8	0.9	1	1	0.87	0.8	1	1	1
	**P38**	1	1	1	1	1	1	1	1	1	1	1	1	1	1	1	1	1	1	1
Late-onset	**P56**	1	1	1	NA	NA	1	0.95	1	1	1	1	1	0.9	0.9	1	1	1	0.9	0.8
Control	**C2**	1	0.75	1	NA	NA	1	0.8	1	1	1	1	1	1	1	1	0.8	1	1	1
	**C3**	NA	0.73	1	NA	NA	NA	NA	1	1	1	1	1	1	1	1	1	1	1	1
	**C9**	1	1	1	NA	NA	NA	0.9	1	1	1	1	1	1	1	1	0.8	1	1	1
	**C10**	1	0.75	1	NA	NA	NA	0.8	1	0.8	0.9	1	1	0.9	0.9	1	1	1	0.87	0.87
Myotubes																				
Juvenile	**P13**	NA	0.4	1	NA	NA	1	0.75	0.65	1	1	1	1	1	1	1	0.5	1	1	1

Adult	**P36**	1	1	1	1	0.9	1	1	0.8	1	1	1	1	1	1	1	1	1	1	1
	**P37**	1	0.5	1	1	0.75	1	0.75	0.75	0.75	1	1	1	1	1	1	1	0.6	1	1
	**P38**	1	1	1	NA	NA	NA	0.5	0.9	1	1	1	1	1	1	1	0.9	1	NA	1
Late-onset	**P56**	1	1	1	NA	NA	1	1	1	1	1	1	1	1	1	1	1	1	1	1
Control	**C2**	1	1	1	NA	NA	1	0.75	1	1	0.9	1	1	1	1	1	1	1	1	1
	**C4**	1	0.9	1	NA	NA	1	0.9	0.8	1	1	1	1	1	1	1	0.75	1	1	1
	**C6**	NA	1	1	1	1	1	1	0.9	1	0.95	0.95	1	1	0.9	0.75	0.8	0.85	0.85	1
	**C9**	NA	1	1	NA	NA	NA	0.75	0.75	1	1	1	1	1	0.9	0.9	0.7	1	0.8	0.9

Range 0 to 1 indicates 0 to 100% methylation. NA = not available

Table S17. DNA methylation analysis of CpGi 36 in skin and skin-derived cells

	CpG →	***1***	***2***	***3***	***4***	***5***	***6***	***7***	***8***	***9***	***10***	***11***	***12***	***13***	***14***	***15***	***16***	***17***
Skin																		
Adult	**P37**	NA	1	1	1	1	1	1	0.75	1	1	1	1	1	0.9	0.9	0.9	0.75
	**P68**	NA	1	1	1	1	1	1	0.8	1	1	1	1	1	1	1	1	1
	**P69**	NA	1	1	1	1	0.9	1	0.9	1	1	1	1	1	1	0.9	0.9	1
Control	**C2**	NA	1	1	1	1	0.8	1	0.8	1	1	1	1	1	1	1	1	0.85
	**C10**	NA	1	1	1	1	0.8	1	0.8	1	1	1	1	1	1	0.75	0.75	1
Skin fibroblasts																		
Juvenile	**P13**	1	1	1	1	0.55	1	1	1	1	0.8	1	1	1	1	1	1	1
Adult	**P36**	1	1	1	1	1	1	0.9	1	1	1	1	1	1	1	0.75	1	1
	**P37**	1	1	1	1	1	1	1	0.9	1	1	1	1	1	1	1	1	0.8
	**P38**	1	1	1	1	1	1	1	0.8	0.9	1	1	1	1	1	0.8	0.9	0.8
Control	**C2**	1	0.7	1	1	1	1	1	0.9	1	1	1	1	1	1	1	0.9	0.9
	**C5**		1	1	1	1	1	1	1	1	1	1	1	1	1	1	1	1
	**C6**	1	1	1	1	1	1	1	0.9	1	1	1	1	1	1	1	1	1
	**C9**	NA	1	0.7	0.7	1	1	1	1	1	1	1	1	1	1	1	1	1

Range 0 to 1 indicates 0 to 100% methylation. NA = not available

Table S18. DNA methylation analysis of CpGi 36 in muscle and muscle-derived cells

	CpG →	***1***	***2***	***3***	***4***	***5***	***6***	***7***	***8***	***9***	***10***	***11***	***12***	***13***	***14***	***15***	***16***	***17***
Muscle																		
Juvenile	**P13**	1	1	1	1	0.8	1	0.9	0.75	1	1	0.9	0.9	0.9	0.55	0.75	0.7	0.75
Adult	**P36**	1	1	1	1	0.9	0.9	0.9	0.75	0.9	0.9	0.9	1	1	0.75	0.85	0.85	1
	**P37**	1	1	1	1	1	1	0.9	0.8	1	1	1	1	1	0.9	1	0.9	0.8
	**P38**	1	1	1	1	1	1	1	0.9	1	1	1	1	1	1	1	1	1
	**P68**	NA	NA	1	1	1	0.8	0.8	0.8	1	1	1	1	1	0.9	0.9	0.8	0.9
Late-onset	**P56**	1	1	1	1	1	1	1	1	1	1	0.8	1	1	0.8	NA	NA	NA
	**P67**	NA	NA	1	0.8	1	1	1	0.8	1	1	1	1	1	1	0.8	0.9	0.9
Control	**C4**	NA	0.5	1	1	1	1	1	0.5	1	1	1	1	1	1	1	0.75	0.63
	**C6**	NA	1	1	1	1	0.85	1	0.75	1	1	1	1	1	1	0.75	0.75	0.9
	**C9**	NA	1	1	1	1	0.8	1	0.9	1	1	1	1	0.9	1	0.75	0.5	0.9
Myoblasts																		
Juvenile	**P13**	1	1	1	1	1	1	1	1	1	1	0.75	1	1	1	0.55	0.8	0.8
Adult	**P36**	1	0.7	1	0.8	1	1	0.9	0.6	1	1	0.8	1	1	0.75	0.7	0.8	0.75
	**P37**	NA	NA	1	1	1	1	1	1	0.95	0.95	0.9	0.9	0.9	0.75	0.9	0.85	0.9
	**P38**	1	1	1	1	1	1	1	1	1	1	1	1	1	1	1	1	1
Late-onset	**P56**	1	1	1	1	1	1	0.8	1	1	1	1	1	1	1	1	1	0.9

Controls	**C3**	1	0.7	1	1	1	1	1	0.7	1	1	1	1	1	1	1	1	0.9
	**C9**	1	0.7	1	1	1	1	1	1	1	1	1	1	1	1	1	0.9	1
	**C10**	1	0.8	1	1	1	1	1	1	1	1	1	1	1	1	1	1	1
Myotubes																		
Juvenile	**P13**	1	1	1	1	1	1	1	1	1	1	0.8	1	1	1	0.6	0.9	0.8
Adult	**P36**	1	1	1	1	1	1	1	0.7	1	1	1	1	1	0.7	0.7	0.8	0.9
	**P37**	1	0.5	0.75	0.75	1	0.55	1	0.55	1	1	0.8	1	1	1	0.7	1	1
	**P38**	1	0.6	1	1	1	1	1	0.75	1	1	1	1	1	0.8	1	0.9	1
Late-onset	**P56**	1	1	1	1	1	1	0.7	1	1	1	1	1	1	1	1	1	0.6
Control	**C2**	1	1	1	1	0.9	0.8	1	0.7	1	1	1	0.9	1	1	1	0.91	
	**C4**	1	0.6	1	1	1	1	1	0.9	1	1	1	1	1	0.8	0.9	1	0.9
	**C6**	1	0.7	1	1	1	1	1	1	1	1	1	1	1	1	1	1	0.6
	**C9**	1	0.7	1	1	1	0.7	1	0.9	1	1	1	1	1	1	1	0.8	0.8

Range 0 to 1 indicates 0 to 100% methylation. NA = not available

Table S19. DNA methylation analysis of CpGi 43 in skin and skin-derived cells

	CpG →	***1***	***2***	***3***	***4***	***5***	***6***	***7***	***8***	***9***	***10***	***11***	***12***	***13***	***14***	***15***
Skin																
Adult	**P37**	0	0	0	0	0	0.15	0	0.05	0.05	NA	NA	0	0	0	0
	**P68**	0	0	0	0	0	0	0	0	0	0	0	0	0	0	0
	**P69**	0	0	0	0	0	0.7	0	0	0	0	0	0	0	0	0
Control	**C2**	0	0	0	0	0.05	0.1	0.05	0	0.05	0	0	0	0.1	0.1	0.1
	**C10**	0	0	0	0.05	0	0.1	0.05	0.05	0.05	0.1	0.1	0.1	0.1	0	0.1
Skin fibroblasts																
Juvenile	**P13**	0	0	0	0	0	0	0	0	0.0.5	0.4	0	0	NA	NA	0.4
Adult	**P36**	0.2	0.25	0.5	0	0.25	0	0	0	0.4	0.5	0	0	0.5	0.05	0.8
	**P37**	0	0	0.05	0	0	0.05	0.05	0.05	0.1	0.1	0.05	0.1	0.1	NA	NA
	**P38**	0	0	0	0	0	0	0	0	0.5	NA	0	0	0	0	0
Control	**C2**	0	0	0.1	0	0.1	0	0.1	0.1	0.1	0.1	0.1	0.1	0.05	0.1	0.3
	**C5**	1	0	0	0	0	0	0.6	0	0.75	0	0	0	0	0	0
	**C6**	0	0	0.4	0	0.3	0	0	NA	0.4	0.4	0.2	NA	0	0.5	0.75
	**C9**	0	0	0	0	0	0	0	0	0	0	0	0	0	0	1

Range 0 to 1 indicates 0 to 100% methylation. NA = not available

Table S20. DNA methylation analysis of CpGi 43 in muscle and muscle-derived cells

	CpG →	***1***	***2***	***3***	***4***	***5***	***6***	***7***	***8***	***9***	***10***	***11***	***12***	***13***	***14***	***15***
Muscle																
Juvenile	**P13**	0.1	0	0.35	0	0.2	0.2	0.4	0.4	0.55	0.25	0	0.25	0.25	0.4	0.75
Adult	**P36**	0	0	0.4	0	0.4	1	0.75	0.5	0.5	1	0.6	0	1	0.5	1
	**P37**	0.25	0	0.5	0	0.4	0.4	0.5	0.5	0.75	0.75	0.5	0	0.75	0.75	0.8
	**P38**	1	0	0.4	0.35	0.4	0.4	0.8	0.45	0.5	0.5	0.5	0.25	0	0.75	1
	**P68**	0	0	0	0	0	0.15	0	0	0	0.2	0	0.25	0.35	0	0.5
Late-onset	**P56**	0.2	0	0.25	0.25	0.25	0.25	0.4	0.4	0.55	0.5	0.5	0.4	0.4	0.4	0.65
	**P67**	0.35	0.25	0.25	0	0.05	0.35	0.35	1	0.75	1	0.45	0.45	0.75	0.1	0.8
Control	**C6**	0.85	0	0.25	0	0.2	0.2	0.75	0.4	0.75	0.55	0.45	0.45	0.45	0	0.85
	**C9**	0.85	0.25	0.2	0	0.2	0.1	0.55	1	0.6	1	0.65	0.25	0.65	0.25	1
Myoblasts																
Adult	**P36**	0	0.2	0	0.5	0.5	0.5	0.5	0.25	0.25	0.25	0.15	0.15	0.75	0.75	0.75
	**P37**	0.15	0	0	0.6	0	0	0	0	0	0	0.5	0	0	0.5	1

	**P38**	0	0	0.25	0.15	0.25	0.15	0.45	0.4	0.5	0.6	0.4	0.5	0.4	0.5	0.75
Late-onset	**P56**	0	0	0.85	NA	NA	0.2	0.75	NA	0	0.2	NA	0	NA	0	0.2
Control	**C2**	0.25	0	0.6	0.25	0.65	0.65	0.78	0.85	0.9	1	0.5	0.75	0.85	0.6	0.9
	**C3**	0.35	0	0.55	0.4	0.55	NA	0.65	0.6	0.6	0.9	0.9	0.6	0.65	0.5	0.6
	**C9**	0.25	0	0.6	0.4	0.5	NA	0.75	0.85	0.85	1	0.75	0.95	0.75	0.65	0.75
	**C10**	0.4	0	0.67	0.25	0.67	0.5	0.75	1	1	1	0.75	0.75	0.6	0.75	0.75
Myotubes																
Juvenile	**P13**	NA	NA	0	0	0	0	0.5	0.2	0.35	0.35	0.4	0.1	0.25	0.25	0.75
Adult	**P36**	0.4	NA	0.45	0.1	0.45	0.5	0.6	0.55	0.65	0.8	0.27	0.25	0.75	0.45	0.8
	**P37**	0.6	0	0.25	0.20	0.5	NA	0.78	1	0.8	1	0.70	0.85	0.35	0.65	0.9
	**P38**	0.05	0	0.15	0.1	0.15	0.2	0.25	0.25	0.5	0.3	0.2	0.25	0.6	0.5	0.75
Late-onset	**P56**	NA	0	0	0.55	0	0.55	0.2	0.6	0.25	0.5	0.6	0.5	0.1	0.6	0.35
Control	**C2**	0.3	0	0.45	0.1	0.75	NA	0.6	0.6	0.75	0.75	0.55	0.7	0.75	0.6	0.8
	**C4**	0.5	0	0.7	0	0.75	0.5	0.8	0.75	1	1	0.75	0.8	0.5	0.75	0.75
	**C6**	0	0	0	0	0.25	0	0	0	0	0	0	0	0	NA	0.3
	**C9**	0.5	0	0.75	0.4	0.55	NA	NA	0.85	1	1	NA	0.75	0.85	1	1

Range 0 to 1 indicates 0 to 100% methylation. NA = not available

Table S21. DNA methylation analysis of CTCF1 in skin and skin-derived cells

	CpG →	*1*	*2*	*3*	*4*	*5*	*6*	*7*	*8*	*9*	*10*	*11*	*12*	*13*	*14*	*15*	*16*	*17*	*18*	*19*	*20*	*21*	*22*	*23*	*24*	*25*
Skin																										
Adult	**P36**	0	0	0	0	0	0	0	0	0	0	0	0	0	0	0	0	0	0	0	0	0	NA	NA	NA	NA
	**P68**	0	0	0	0	0	0	0	0	0	0	0	0	0	0	0	0	NA	NA	NA	NA	NA	NA	NA	NA	NA
Control	**C2**	0	0	0	0	0	0	0	0	0	0	0	0	0	0	0	0	0	0	0	0	0	NA	NA	NA	NA
	**C10**	0	0	0	0	0	0	0	0	0	0	0	0	0	0	0	0	0	0	0	0	0	NA	NA	NA	NA
Skin fibros																										
Juvenile	**P13**	NA	0.15	0.25	0.1	0.1	0.1	0.05	0.1	0.1	0.75	0.25	0.1	0.25	0.3	0.5	0.2	0.1	0.1	0.1	0.1	0	0.05	0.15	0.1	0.15
Adult	**P36**	NA	0.1	0.1	0.1	0.05	0.05	0.05	0.05	0.05	0.05	0.05	0.1	0.1	0.15	0.1	0.05	0	0	0	0	0	0	0	0	0
	**p37**	NA	NA	0	0	0.1	0.1	0	0	0	0.15	0.2	0.4	0.1	0.15	0.1	0	0.1	0.1	0.25	0.25	0.05	0.05	0	0	0
	**P38**	0	0	0	0	0	0	0	0	0	0	0	0.4	0.35	0.4	0	0	0	0	0	0	0	0	0	0	0.4
Control	**C2**	0	0	0	0	0	0	0	0	0	0	0	0	0	0	0	0	0	0	0	0	0	0	0	0	0
	**C5**	0	0	0	0	0	0	0	0	0	0	0	0	0	0	0	0	0	0	0	0	0	NA	NA	NA	NA
	**C6**	NA	0	0	0	0	0	0	0	0	0	0	0	0	0	0	0	0	0	0	0	0	NA	NA	NA	NA
	**C9**	0	0	0	0	0	0	0	0	0	0	0	0	0	0	0	0	0	0	0	0	0	NA	NA	NA	NA

Range 0 to 1 indicates 0 to 100% methylation. NA = not available, Skin fibros= skin fibroblasts

Tables S22. DNA methylation analysis of CTCF1 in muscle and muscle-derived cells

	CpG →	*1*	*2*	*3*	*4*	*5*	*6*	*7*	*8*	*9*	*10*	*11*	*12*	*13*	*14*	*15*	*16*	*17*	*18*	*19*	*20*	*21*	*22*	*23*	*24*	*25*
Muscle																										
Juvenile	**P13**	0.2	0.1	0.1	0.2	0.1	0.1	0	0.15	0.2	0.15	0.2	0.25	0.1	0.25	0.25	0.4	0	0	0	NA	NA	NA	NA	NA	NA
Adult	**P36**	0	0	0	0	0	0	0	0.1	0	0	0	0	0	0.1	0.1	0.1	0	0	0	NA	NA	NA	NA	NA	NA
	**P37**	0.1	0.1	0.05	0.1	0	0.05	0.05	0.05	0.05	0.1	0.2	0.2	0.2	0.2	0.2	0.2	0	0	0	NA	NA	NA	NA	NA	NA
	**P38**	0.1	0.1	0.1	0.1	0.1	0	0.1	0.1	0.1	0.2	0.2	0.2	0	0	0	0	0	0	0	NA	NA	NA	NA	NA	NA
	**P68**	NA	0.2	0.2	0	0	0	0	0	0	0	0	0	0.2	0	0	0	0	0.2	NA	NA	NA	NA	NA	NA	NA
Late-onset	**P56**	0	0.05	0.05	0	0	0	0	0	0	0.1	0.1	0.1	0	0	0	0.1	0	0	0	NA	NA	NA	NA	NA	NA
	**P67**	NA	0.05	0.15	0	0	0	0	0	0	0	0	0	0	0	0	0	0	0	NA	NA	NA	NA	NA	NA	NA
Control	**C4**	0	0	0	0	0	0	0	0	0	0	0	0	0	0	0	0	0	0	0	NA	NA	NA	NA	NA	NA
	**C6**	0	0	0	0	0	0	0	0	0	0	0	0	0	0	0	0	0	0	0	NA	NA	NA	NA	NA	NA
	**C9**	0	0	0	0	0	0	0.1	0	0	0	0	0	0	0	0	0	0	0	0	NA	NA	NA	NA	NA	NA
Myoblasts																										
Juvenile	**P13**	NA	0.25	0.4	0.2	0.1	0.1	0	0.15	0.15	0.75	0.25	0.25	0.25	0.4	0.6	0.2	0.1	0.1	0.1	0.1	0	0.1	0.15	0.1	0.1
Adult	**P36**	NA	NA	NA	0	0	0	0	0	0.05	0.05	0.05	0.1	0	0	0	0.15	0	0	0	0	0	0	0	0	0
	**P37**	0.4	0.1	0.4	0.8	0	0.8	0	0	0	1	0	1	1	1	0.75	1	0	0	0	0	0	0	0	0	0.85

	**P38**	0.15	0.15	0	0	0	0	0	0	0	0	0	0	0	0	0	0	0	0	0	0	0	0	0	0	0
Late-onset	**P54**	NA	0.25	0.05	0.25	0.05	0	0	0	0	0.2	0.1	0.2	0.1	0.1	0.35	0.2	0	0	0	0	0	0	0.05	0.05	0.05
Control	**C2**	NA	0	0	0	0	0	0	0	0	0	0	0	0	0	0	0	0	0	0	0	0	0	0	0	0
	**C3**	NA	0	0	0	0	0	0	0	0	0	0	0	0	0	0	0	0	0	0	0	0	0	0	0	0
	**C9**	NA	0	0	0	0	0	0	0	0	0	0	0	0	0	0	0	0	0	0	0	0	0	0	0	0
	**C10**	NA	0	0	0	0	0	0	0	0	0	0	0	0	0	0	0	0	0	0	0	0	0	0	0	0
Myotubes																										
Juvenile	**P13**	NA	0.2	0.25	0.2	0.1	0.1	0.1	0.15	0.15	0.4	0.25	0.2	0.25	0.25	0.2	0.4	0	0.1	0.2	0	0	0	0.1	0.1	0.1
Adult	**P36**	NA	0.15	0.1	0.15	0.1	0.1	0	0.15	0.15	0.2	0.15	0.2	0.1	0.15	0.15	0.2	0	0	0	0	0	0	0	0	0
	**P37**	NA	1	1	0.4	0.1	0.1	0.05	0.2	0.2	0.25	0.2	0.5	0.25	0.25	0.25	0.65	0	0.05	0	0.05	0.05	0.1	0.15	0.1	0.4
	**P38**	0.15	0.15	0	0	0	0	0	0	0	0	0	0	0	0	0	0	0	0	0	0	0	0	0	0	0
Late-onset	**P54**	NA	0.2	0.05	0.6	0.05	0	0.05	0.05	0.05	0.05	0.05	0.1	0.1	0.6	0.15	0	0	0	0	0.1	0	0	0	0	0
Control	**C2**	NA	0	0	0	0	0	0	0	0	0	0	0	0	0	0	0	0	0	0	0	0	0	0	0	0
	**C4**	NA	0	0	0	0	0	0	0	0	0	0	0	0	0	0	0	0	0	0	0	0	0	0	0	0
	**C9**	NA	0	0	0	0	0	0	0	0	0	0	0	0	0	0	0	0	0	0	0	0	0	0	0	0
	**C6**	NA	0	0	0	0	0	0	0	0	0	0	0	0	0	0	0	0	0	0	0	0	0	0	0	0

Range 0 to 1 indicates 0 to 100% methylation. NA = not available

Table S23. CTG expansion sizes in blood and muscle of the patients providing a muscle biopsy

	Blood progenitor	*Blood modal*	*Muscle progenitor*	*Muscle modal*
Juvenile P13	286	445	691	736
Adult P36	180	338	208	335
Adult P37	199	374	469	788
Adult P38	115	130	135	207
Adult P68	322	619	351	758
Late-onset P56	300	381	NA	NA
Late-onset P67	108	246	128	512

NA = not available

Table S24. DNA methylation analysis of CTCF2 in skin and skin-derived cells

		CpG→	*1*	*2*	*3*	*4*	*5*	*6*	*7*	*8*	*9*	*10*	*11*
Skin													
	Adult	**P37**	NA	NA	NA	0	0	0	0	0	0	0	0
		**P68**	NA	NA	NA	0	0	0	0	0	0	0	0
		**P69**	NA	NA	NA	0	0	0	0	0	0	0	0
	Control	**C2**	NA	NA	NA	0	0	0	0	0	0	0	0
		**C10**	NA	NA	NA	0	0	0	0	0	0	0	0
Skin fibroblasts													
	Juvenile	**P13**	NA	0	0	0	0	0	0	0	0	0	0
	Adult	**P36**	NA	0	0	0	0	0	0	0	0	0	0
		**P38**	0	0	0	0	0	0	0	0	0	0	0
	Control	**C2**	NA	0	0	0	0	0	0	0	0	0	0
		**C5**	0	0	0	0	0	0	0	0	0	0	0
		**C6**	0	0	0	0	0	0	0	0	0	0	0
		**C9**	0	0	0	0	0	0	0	0	0	0	0

Range 0 to 1 indicates 0 to 100% methylation. NA = not available

Table S25. DNA methylation analysis of CTCF2 in muscle and muscle-derived cells

	CpG →	1	2	3	4	5	6	7	8	9	10	11
Muscle												
Juvenile	**P13**	NA	NA	NA	0	0	0	0	0	0	0	0
Adult	**P36**	NA	NA	NA	0	0	0	0	0	0	0	0
	**P37**	NA	NA	NA	0	**0**	0	0	0	0	0	0
	**P38**	NA	NA	NA	0	0	0	0	0	0	0	0
	**P68**	NA	NA	NA	0	0	0	0	0	0	0	0
Late-onset	**P56**	NA	NA	NA	0	0	0	0	0	0	0	0
	**P67**	NA	NA	NA	0	0	0	0	0	0	0	0
Control	**C4**	NA	NA	NA	0	0	0	0	0	0	0	0
	**C6**	NA	NA	NA	0	0	0	0	0	0	0	0
	**C9**	NA	NA	NA	0	0	0	0	0	0	0	0
Myoblasts												
Juvenile	**P13**	NA	0	0	0	0	0	0	0	0	0	0
Adult	**P36**	NA	0	0	0	0	0	0	0	0	0	0
	**P37**	NA	0	0	0	0	0	0	0	0	0	0
	**P38**	NA	0	0	0	0	0	0	0	0	0	0
Late-onset	**P56**	NA	0	0	0	0	0	0	0	0	0	0
Control	**C2**	0	0	0	0	0	0	0	0	0	0	0
	**C3**	0	0	0	0	0	0	0	0	0	0	0
	**C9**	0	0	0	0	0	0	0	0	0	0	0
	**C10**	0	0	0	0	0	0	0	0	0	0	0
Myotubes												
Juvenile	**P13**	NA	0	0	0	0	0	0	0	0	0	0
Adult	**P36**	NA	0	0	0	0	0	0	0	0	0	0
	**P37**	NA	0	0	0	0	0	0	0	0	0	0
	**P38**	NA	0	0	0	0	0	0	0	0	0	0
Late-onset	**P56**	NA	0	0	0	0	0	0	0	0	0	0
Control	**C2**	0	0	0	0	0	0	0	0	0	0	0
	**C4**	0	0	0	0	0	0	0	0	0	0	0
	**C9**	0	0	0	0	0	0	0	0	0	0	0
	**C6**	0	0	0	0	0	0	0	0	0	0	0

Range 0 to 1 indicates 0 to 100% methylation. NA = not available
